# Robust Full‐Surface Bonding of Substrate and Electrode for Ultra‐Flexible Sensor Integration

**DOI:** 10.1002/adma.202417590

**Published:** 2025-02-18

**Authors:** Masahito Takakuwa, Daishi Inoue, Lulu Sun, Michitaka Yamamoto, Shinjiro Umezu, Daisuke Hashizume, Toshihiro Itoh, Kenjiro Fukuda, Takao Someya, Tomoyuki Yokota

**Affiliations:** ^1^ Graduate School of Engineering Institute of Engineering Innovation The University of Tokyo 7‐3‐1 Hongo Bunkyo‐ku Tokyo 113‐8656 Japan; ^2^ Department of Electrical Engineering and Information Systems The University of Tokyo 7‐3‐1 Hongo Bunkyo‐ku Tokyo 113‐8656 Japan; ^3^ Center for Emergent Matter Science (CEMS) RIKEN 2‐1 Hirosawa Wako Saitama 351‐0198 Japan; ^4^ Thin‐Film Device Laboratory RIKEN 2‐1 Hirosawa Wako Saitama 351‐0198 Japan; ^5^ Department of Precision Engineering Graduate School of Engineering The University of Tokyo Tokyo 113‐8656 Japan; ^6^ Department of Modern Mechanical Engineering Waseda University 3‐4‐1 Okubo Shinjuku‐ku Tokyo 169–8555 Japan

**Keywords:** flexible integration method for flexible electronics, hybrid direct bonding method, low‐temperature gold bonding, low‐temperature parylene fusion bonding

## Abstract

The integration of multiple flexible electronics is crucial for the development of ultra‐flexible wearable and implantable devices. To fabricate an integrated system, robust and flexible bonding throughout the connection area, irrespective of the electrode or substrate, is needed. Conventional methods for flexible direct bonding have primarily been confined to metal electrodes or substrate‐only bonding due to varying material properties. Consequently, the mechanical and electrical properties of the connections deteriorate based on their shape and size. This study introduces a bonding technique for wearable electronics, achieving strong, flexible connections between materials like gold and parylene at a low temperature (85 °C). This hybrid direct bonding method ensures strong bonding across both the Au electrode and parylene substrate within electronic interconnections. Additionally, a 3D‐stacked flexible structure that maintains robustness and high flexibility without an adhesive layer is successfully developed. An ultrathin photoplethysmography sensor developed by stacking an ultrathin organic photodetector atop an organic light‐emitting diode is demonstrated. Unlike traditional methods requiring adhesives or high pressure, this approach maintains flexibility essential for deformation, withstanding bending at a radius of 0.5 mm. The technique's robustness suggests promising applications in durable, ultra‐flexible electronics integration.

## Graphical abstract

A direct hybrid bonding method that enables both gold and parylene bonding without adhesives is developed. This technique achieves full‐surface direct bonding of electrodes and substrates in flexible electronic connections. The method offers high flexibility, stable mechanical durability, and high‐resolution interconnections, accommodating varying electrode shapes and sizes in flexible electronic applications.

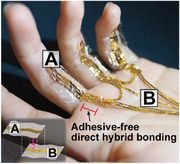

## 1 Introduction

The integration of a variety of flexible electronic components, such as sensors, transmitters, and energy harvesters, onto assorted film substrates is essential for the advancement of cutting‐edge wearable and implantable devices.^[^ ^1^, ^2^, ^3^ ^]^ Increasing the number of electronic components per unit area is crucial for enhancing device miniaturization and functionality.^[^ ^1^ ^]^ Given the ability to miniaturize flexible electronics to microscale dimensions,^[^ ^4^ ^]^ there is a corresponding need for microscale resolution in bonding and wiring technologies to facilitate integration. Furthermore, these electronics must endure continual mechanical stresses as they conform to the dynamic contours of the skin or internal organs.^[^ ^5^, ^6^, ^7^ ^]^ Therefore, it is imperative to develop a bonding strategy that not only minimizes stress concentration at the electrodes but also provides exceptional flexibility, allowing the device to seamlessly integrate and adhere to the human body.

Conventional methods for bonding flexible electronics typically employ conductive intermediate layers such as Ag paste,^[^ ^7^ ^]^ liquid metals,^[^ ^8^ ^]^ and anisotropic conductive film (ACF) tape.^[^ ^9^, ^10^ ^]^ ACF tape, in particular, has proven effective for creating robust bonds, capable of connecting joints across various substrates and metal electrodes, irrespective of their material properties. By optimizing the balance of conductive particles and insulating adhesives, ACF tape has achieved resolutions down to 10 µm.^[^ ^10^ ^]^ Post‐processing treatments, including ultraviolet irradiation, heating (≈200 °C), and pressurization, are utilized to ensure optimal conductivity. However, the incorporation of adhesives, while beneficial for connectivity, must be carefully calibrated to prevent significant reductions in the electronics' flexibility (Table S1, Supporting Information).

The most effective strategy for maintaining flexibility while bonding flexible electronics is to avoid using adhesive layers. Since organic semiconductor materials generally have a heat resistance of ≈100 °C, direct bonding processes that operate below this temperature are predominantly employed for integrating these electronics (Table S1, Supporting Information). These processes can be divided into two main categories. The first method involves direct bonding of film substrates using materials with Young's moduli on the order of kilopascals to megapascals, such as dimethylpolysiloxane or styrene‐ethylene‐butylene‐styrene (SEBS).^[^ ^7^, ^11^ ^]^ These materials adhere closely when they come into contact due to van der Waals forces, ensuring the contact between the electrodes and forming a conductive pathway. By incorporating metal particles within SEBS with a carefully controlled distribution, the interface achieves cohesive properties with a bonding resolution down to 100 µm.^[^ ^11^ ^]^ The second method focuses on the direct bonding of metal electrodes using either pressurization or plasma treatment. Cold welding, for example, applies significant pressure (ranging from tens to hundreds of megapascals) to disrupt oxide layers and other impurities on metal surfaces. This allows atoms to diffuse through the interface due to plastic deformation, creating atomic‐level bonds between the metals.^[^ ^12^, ^13^ ^]^ Surface‐activated bonding (SAB) is another technique, which involves plasma irradiation to clean the surfaces by removing contaminants and oxygen layers without pressing the surfaces together; this method achieves low‐temperature direct bonding.^[^ ^14^, ^15^, ^16^ ^]^ While conventional SAB requires smooth surfaces (with the root mean square (RMS) of surface roughness less than several nanometers) and high contact pressures (tens or hundreds of megapascals) to ensure atomic‐level bonding, recent developments have demonstrated nearly pressure‐less bonding by preparing ultra‐smooth, flat surfaces (RMS surface roughness <1 nm).^[^ ^15^, ^17^ ^]^ Furthermore, the direct bonding of rough gold electrodes fabricated on polymer substrates (RMS surface roughness ≈7 nm) has been successfully performed by optimizing the plasma gas source and using a flexible substrate.^[^ ^18^ ^]^ This adapted SAB approach, employing water vapor, has achieved a fine bonding resolution of 10 µm. As this method does not apply pressure, it significantly reduces the risk of damaging the delicate thin‐film substrates and individual layers, making it an ideal technique for assembling flexible electronics on thin films.

However, existing direct bonding technologies have yet to provide a solution that combines high flexibility, robust bonding across the entire interface (including both polymer substrates and metal electrodes), and a conductive bonding resolution under 5 µm. Conventional direct bonding methods, typically operating from room temperature to below 100 °C, are constrained to either metal or polymer bonding due to the stark differences in the material properties of metals and polymers. Metals and polymers not only differ in melting points but also in the types of bonds they form—metallic and chemical, respectively. These differences make it challenging to achieve high‐resolution and robust full‐surface bonding in the interconnection regions of flexible electronics, where disparate materials coexist. Additionally, partial direct bonding within these areas often results in stress concentration from deformation, leading to decreased bonding strength and increased contact resistance, which are significant challenges in integration technologies.

In this study, we developed a hybrid direct bonding method capable of simultaneously bonding both Au and the paraxylene polymer (commonly known as parylene) at a process temperature of 85 °C. By incorporating a pretreatment process involving plasma treatment and the plasticizing effects of water, we managed to lower the bonding temperature for parylene by more than 50 °C compared to previous efforts, keeping it well below the thermal threshold of organic electronics (Figure S1, Supporting Information). This method facilitated robust atomic‐level bonding of gold electrodes and direct bonding of parylene substrates at the same temperature. As a result, our technology allows for the seamless bonding of the entire interface of flexible electronics, including both polymer substrates and metal electrodes, without the need for adhesives. This ensures stable mechanical durability and high‐resolution interconnections, irrespective of the shape and size of the electrodes. The minimum electrode width and spacing achieved were less than 5 µm, with the smallest bonding area measuring less than 50 µm × 50 µm. The contact resistance in the electrode‐bonding region is 0.33 m Ω cm^−^^2^. After enduring 10 000 bending cycles, the electrical resistance displayed a minimal change of 3% (from 4.5 to 4.4 Ω). This hybrid direct bonding method has demonstrated a bonding strength greater than ten times that achieved by the previously developed customized SAB method using water‐vapor plasma assisted bonding (WVPAB), which was limited to bonding only gold electrodes.^[^ ^18^ ^]^ Furthermore, the method enabled the development of an ultrathin photoplethysmography (PPG) sensor by integrating ultrathin organic photodetectors (OPDs) and organic light‐emitting diodes (OLEDs). The total thickness of the assembled system is simply the sum of its individual components, showcasing exceptional flexibility with a bending radius as small as 0.5 mm. This bonding approach thus stands out as a highly promising integration technology, combining superior flexibility, mechanical durability, and precise bonding resolution—qualities indispensable for the advancement of stacked flexible and implantable electronic devices.

## 2 Results

### 2.1 Low‐Temperature Bonding via Plasma‐Activation and Water‐Plasticizer Effect

**Figure 1:**
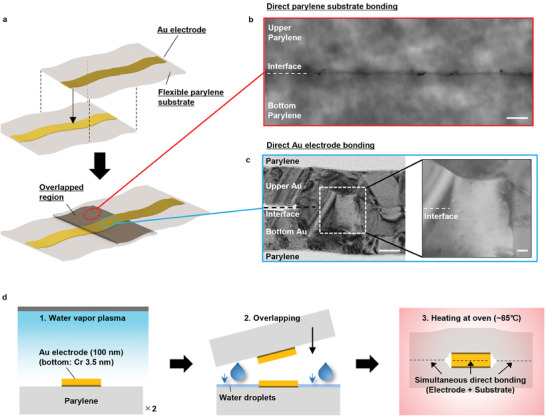
Ultra‐flexible and robust integration through hybrid direct bonding. a) Schematic of direct bonding between thin‐film materials. Each substrate‐to‐substrate and electrode‐to‐electrode bond aligns precisely, facilitating direct attachment across the overlapping region without adhesive materials. b) Cross‐sectional STEM image of the parylene bonding region showing strong bonding, characterized by higher electron density compared to the inner parylene films. Scale bar: 100 nm. c) Cross‐sectional TEM image of the gold bonding region. Left image scale bar: 50 nm; right expanded image scale bar: 10 nm. d) Process flow for hybrid direct bonding: 1) Surface activation of all bonding regions using water vapor plasma; 2) Bringing treated bonding surfaces into contact followed by the injection of a small amount of water; 3) Applying low‐temperature heating to the overlapped samples in an oven.

This section introduces a novel direct bonding technique termed low‐temperature bonding via plasma activation and water plasticizer effect (LBPW) (**Figure** **1**). Conventional parylene thermal bonding typically requires temperatures exceeding 160 °C (Figure S1, Supporting Information). On the other hand, we successfully reduced the bonding temperature to just 85 °C by utilizing plasma and droplet‐based pretreatments. Additionally, the combination of low‐temperature heating necessary for parylene bonding and the water vapor plasma treatment ensures robust, atomic‐level direct bonding of gold. Consequently, the LBPW method enables simultaneous bonding of the entire bonding surface, including the substrate (thin‐film parylene) (Figure 1b) and the electrode regions (Au electrodes) (Figure 1c), regardless of the proportion of polymer substrate to electrode area on the bonding surface. This method ensures highly reliable and strong direct bonding. During the LBPW process (Figure 1d; Figure S2, Supporting Information), water vapor plasma treatment (50 W, H_2_O gas: 12 sccm, 40 s, RIE mode) is applied to the bonding surfaces, followed by the immediate contact of these treated surfaces under ambient conditions, resulting in achieved partial gold‐to‐gold bonding. At this stage, direct bonding between the parylene substrates did not occur, but substantial interfacial adhesion was achieved due to surface modification. This process was followed by the injection of deionized water into the overlapping regions of the parylene substrate. Subsequent low‐temperature heating at 85 °C in an oven then facilitated robust direct bonding of both the gold electrodes and parylene.

Cross‐sectional views of the samples bonded using the LBPW method were examined using scanning transmission electron microscopy (STEM) and transmission electron microscopy (TEM). The images revealed a continuous, highly electron‐dense region, ≈4.9 nm thick, across the entire parylene‐substrate bonding interface, with no voids present, indicating a homogeneous bond. This bonding interface exhibited higher crystallinity than the interior regions of the parylene films (Figure 1b). Additionally, within the Au electrode bonding area, metal crystals were observed growing through the interface, effectively eliminating the visibility of the original boundary. Energy‐dispersive X‐ray spectroscopy (EDX) analysis performed via a line scan on both the interior of the gold bonding region and at the bonding interface showed no variation in atomic composition between the two areas (Figure S3, Supporting Information). This uniformity indicates that the upper and lower gold electrodes merged seamlessly due to the plasma surface treatment and subsequent heating, creating a highly robust atomic‐level bond (Figure 1c). Compared to the preheat treatment conditions, the region where the interface disappeared was notably larger, and there was a smooth metallic crystal contrast change from upper to bottom regardless of the interface. suggests a more extensive occurrence of atomic diffusion bonding than previously achieved with the conventional WVPAB method (Figure S4, Supporting Information).

### 2.2 Low‐Temperature Bonding of Parylene Polymer Substrate via an LBPW Process

The bonding effectiveness of parylene films was explored under a variety of conditions using different plasma gas types (water vapor, oxygen, and argon), levels of water content, and heating methods. These experiments were quantitatively assessed through a series of bonding tests, followed by a 90° delamination test conducted under underwater immersion to evaluate the potential for interfacial delamination or substrate failure (Figure S5, Supporting Information). Notably, samples subjected to just water application and heating without plasma treatment consistently exhibited interface failure. In contrast, samples that underwent plasma treatment, regardless of the gas used, predominantly showed substrate failure during delamination tests, indicating robust bonding (Figures S6a,S7a, Supporting Information).

For the simultaneous hybrid direct bonding of Au electrodes and parylene substrates, water vapor plasma treatment was selected due to its effectiveness in ensuring the direct bonding of Au electrodes as demonstrated in prior studies.^[^ ^18^ ^]^ To determine the allowable atmospheric exposure time post‐plasma treatment, samples were exposed to ambient conditions for durations ranging from 2 to 25 h. Samples subsequently heated at 85 °C for 4 h after injecting deionized (DI) water into the interface showed varying results: those exposed for 6 h exhibited substrate failure, while those exposed for 25 h showed signs of interfacial delamination, indicating optimal bonding achieved with at least 6 h of post‐plasma exposure (Figures S6b,S7b, Supporting Information).

Further optimization of the bonding conditions involved adjusting the quantity of water deposited. The water droplet amounts ranged from 0 to 5.0 µL/100 mm^2^ to find the optimal volume for effective bonding. Successful bonding characterized by substrate failure was consistently observed with water deposition amounts of 0.5, 1.0, and 3.0 µL (Figures S6c,S7c, Supporting Information). In contrast, there was a 0% bonding rate when no water was applied. Samples with a water deposition of 5.0 µL often failed to bond, resulting in a lower bonding yield. Following these findings, we proceeded to refine the optimal heating temperature for the parylene bonding process.

**Figure 2:**
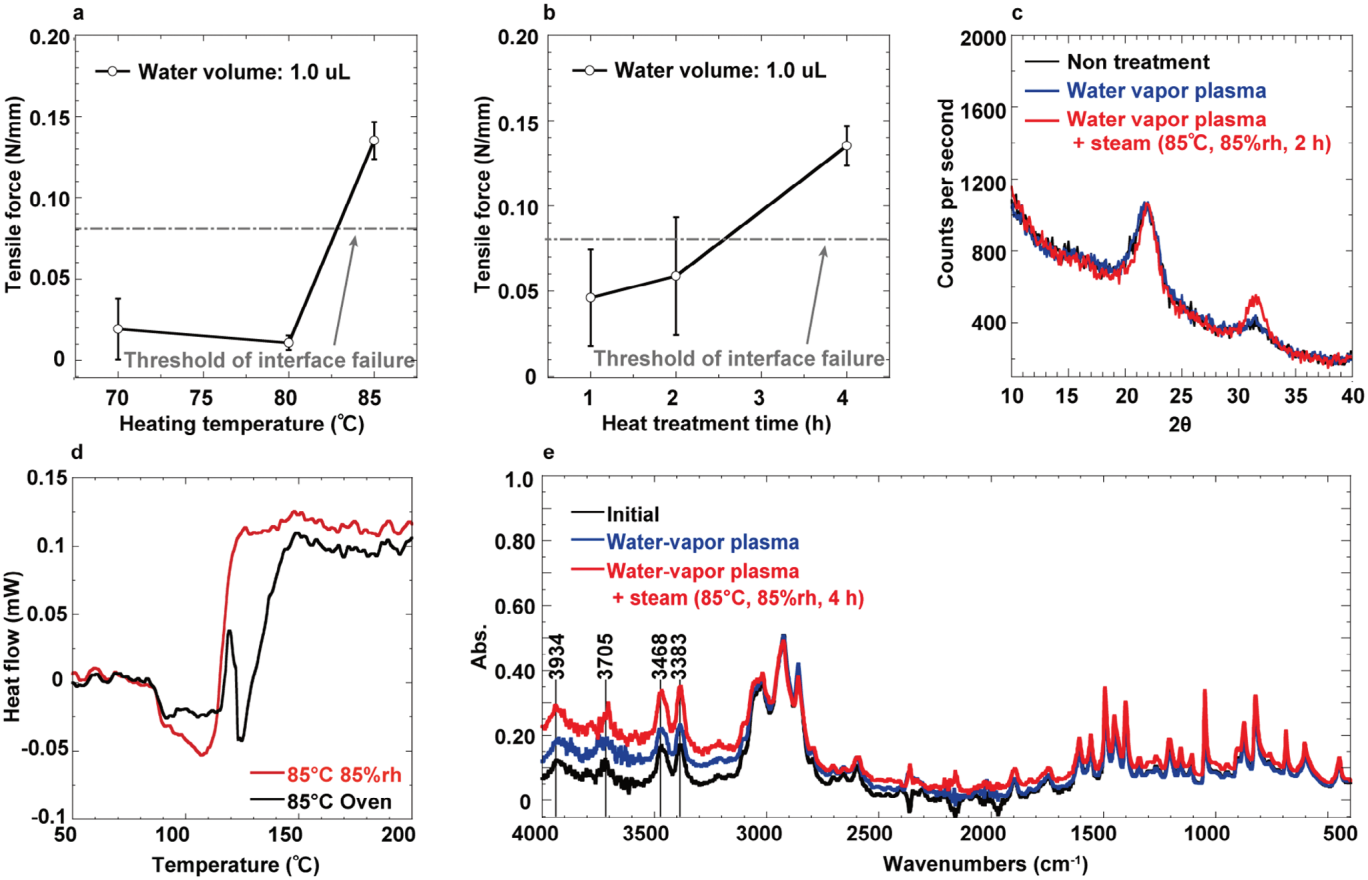
Parylene direct bonding conditions and mechanism analysis at low temperature. Variations in bonding outcomes associated with different heat treatments are shown. Tensile test results exceeding 0.08 N/mm indicate substrate failure, while results below 0.08 N/mm suggest interface delamination. a) Variation in direct bonding results across different heating temperatures for 4 h. (mean ± standard deviation (SD), *n =* over 6) b) Variation in direct bonding results across different durations at 85 °C. (mean ± SD, *n =* over 6) c) Changes in crystallinity under steam treatment with water vapor plasma, as determined by XRD measurements. d) Comparison of the thermal histories of parylene films prepared by various heating methods, analyzed using DSC measurements. e) Surface analysis conducted using FTIR measurements at each step of LBPW bonding.

Experiments were conducted under controlled plasma conditions with a fixed heating duration of 4 h. At a heating temperature of 85 °C, samples exhibited substrate failure, while those heated below 80 °C showed interfacial failure (**Figure** **2**a; Figure S7d, Supporting Information). Therefore, the minimum heating temperature for achieving direct parylene bonding using the LBPW process was established as 85 °C. The effect of heating time was also assessed, with experiments conducted at a constant plasma condition and heating temperature (85 °C). Results indicated that the parylene bonding rate reached 100% after heating for more than 4 h (Figure 2b; Figure S7e, Supporting Information). Additionally, the LBPW method demonstrated superior water stability compared to hydrogen bonding with plasma treatment (Movie S1, Supporting Information), as the LBPW‐bonded samples remained intact after eight days of water immersion (Movie S2, Supporting Information). This study primarily utilized parylene‐SR polymer; however, direct bonding was also achieved with parylene C polymer using the LBPW process (Figure S8, Supporting Information).

The versatility of chemical vapor deposition (CVD) allows parylene films to be deposited onto a variety of materials and complex geometries, enabling heterogeneous material bonding through parylene mediation. For these experiments, a 1.3‐µm‐thick parylene film was deposited onto a 1‐µm‐thick polyethylene terephthalate (PET) film and a 5‐µm‐thick polyimide film. The samples were then directly bonded using the LBPW method, with material combinations detailed in Table S2 (Supporting Information). Direct bonding was confirmed for all combinations, each involving polymers of different types and thicknesses. The observed fracture modes indicated that the failure was not adhesive but involved delamination of the parylene layer from the PET and polyimide surfaces (Figures S9,S10, Supporting Information).

### 2.3 Change in Material State Due to LBPW Treatment

To quantitatively evaluate the impact of the LBPW on parylene films, various analyses were performed. Instead of the conventional method of water dropping and oven heating post water‐vapor plasma treatment, controlled heat and moisture were applied using a steam chamber (85 °C, 85% rh). Initially, tensile tests were conducted on three types of unbonded single parylene films: untreated, steam‐treated, and oven‐heated. The results indicated a significant increase in shear strength of over 20% for the steam‐treated parylene films compared to the untreated ones. Additionally, steam‐treated films exhibited ≈7% higher shear force and less variation than those subjected to oven heating (Figure S11, Supporting Information). To explore the underlying changes in mechanical properties resulting from the pretreatment, we investigated the modifications in crystallinity of the parylene films using X‐ray diffraction (XRD), cross‐sectional scanning transmission electron microscopy (STEM), and differential scanning calorimetry (DSC). XRD analysis revealed an increased crystalline peak at 2*θ* = 31° following heat treatment, indicative of enhanced crystallinity in comparison to untreated films (Figure 2c). STEM cross‐sections showed that untreated parylene films primarily displayed noncrystalline, white regions (Figure S12a, Supporting Information). In contrast, the treated films demonstrated a notably increased black crystalline region, especially in areas exposed to plasma treatment, which displayed exceptionally high crystallinity (Figure S12b, Supporting Information).

Further, DSC analysis provided insights into the thermal behavior of these films. For oven‐heated parylene, an exothermic peak appeared due to the crystallization of the amorphous regions following the glass transition, as crystallization was incomplete at 85 °C over 21 h (Figure 2d). Conversely, steam heating at the same temperature did not show this exothermic peak. This finding suggests that the LBPW‐treated parylene films achieved higher crystallinity than those merely heated. Therefore, the DSC analysis confirmed that the combined influence of moisture and heat in the LBPW treatment promoted more extensive crystallization of the parylene films compared to heating alone.

Atomic force microscopy (AFM) was employed to evaluate the physical changes to the bonding surfaces resulting from the LBPW treatment. The analysis indicated that the RMS roughness of parylene decreased slightly from 5.1 to 5.0 nm, representing a reduction factor of 0.98, while that of gold increased marginally from 5.3 to 5.5 nm, with an increase factor of 1.04 (Figure S13, Supporting Information). Consequently, the impact of water vapor plasma treatment on surface roughness was determined to be minimal.

Chemical modifications of the surface due to LBPW treatment were investigated using Fourier transform infrared spectroscopy (FTIR) and X‐ray photoelectron spectroscopy (XPS). FTIR analysis revealed no significant change in the chemical state of the surfaces, as evidenced by the absence of peak shifts pre‐ and post‐treatment (Figure 2e). However, an increase in peak intensity within the hydrogen bonding region (3200–4000 cm^−1^) was noted after treatment (Table S3, Supporting Information), suggesting modifications at the molecular level.

The nature of the bonding mechanism was further examined by forcibly peeling the bonded interface and analyzing the separated surfaces using XPS (Figure S14, Supporting Information). This procedure revealed a distinct differentiation between a cloudy, bonded area and a clear, unbonded region (Figure S14a,b, Supporting Information). Chemical bonding states at these interfaces were compared, with XPS results showing identical binding peaks on both surfaces (Figure S15 and Table S4, Supporting Information), indicating consistent chemical characteristics across the bonded and unbonded areas.

Elemental analysis of the bonded region revealed a decrease in the carbon content and an increase in oxygen compared to the unbonded region. Additionally, curve fitting of the C 1s XPS signal in the cloudy regions showed that 1.1% of the carbon was involved in C═O bonds and 0.6% in C─O─O bonds. These proportions were slightly higher than those in the unbonded parts (Figure S16 and Table S5, Supporting Information). This suggests that the OH groups crucial for interfacial adhesion underwent dehydration upon heating, leading to the formation of these specific chemical bonds.

### 2.4 Mechanical and Electric Properties of LBPW Bonding

**Figure 3:**
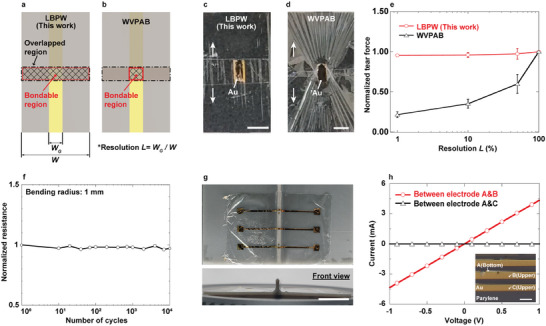
Mechanical and electrical characteristics of 2‐µm‐thick parylene electrodes bonded using LBPW bonding. a) Schematic showing the bondable region for direct bonding via LBPW, illustrating bonding across the entire overlapped region. b) Schematic showing the bondable region for WVPAB bonding. c) Image of the bonded thin‐film sample using the LBPW method during a tensile test. Scale bar: 5 mm. d) Image of the bonded thin‐film sample using the WVPAB method during a tensile test. Scale bar: 5 mm. e) Comparison of mechanical durability between the LBPW and WVPAB bonding methods across various resolutions (defined as the ratio of electrode width to substrate width). (mean ± SD, *n* of LBPW = 3, *n* of WVPAB = 2) f) Cyclic bending durability test of the LBPW bonding method. g) Conformability test of the bonding region when applied to a 0.5 mm bump. Scale bar: 10 mm. h) Current‐voltage characteristics of the bonded thin‐film wires with a 5 µm L/S ratio via LBPW bonding. The inset is an optical microscope image of the bonded sample with 5 µm L/S. Scale bar: 10 µm.

Tensile tests were performed to evaluate the mechanical durability of hybrid direct bonding between parylene substrates and Au electrodes using the LBPW. These tests varied the width ratio of the gold electrodes to parylene substrates from 50% to 1% (**Figure** **3**a). For comparative analysis, identical tensile tests were conducted using WVPAB (Figure 3b). The LBPW approach facilitated the bonding of both the gold electrodes and the parylene polymer substrate, allowing stress to be evenly distributed across the entire bonded region (Figure 3c). Conversely, the WVPAB method bonded only the gold electrodes, resulting in concentrated deformation stress on the central gold electrode during tensile testing (Figure 3d). The findings indicate that samples bonded using WVPAB exhibited reduced shear strength and wire width due to the exclusive bonding of the Au electrode region. In contrast, LBPW achieved direct bonding of both the parylene substrate and Au electrode, maintaining constant shear strength regardless of wire width variation (Figure 3e).

This distinction in mechanical bonding strength is particularly significant in the fabrication of multilayer structures for flexible electronics applications. When comparing 3D‐stacked flexible bonding samples prepared using WVPAB and LBPW (Figures S17,S18, Supporting Information), the WVPAB samples displayed numerous wrinkles in the overlapping substrate region, due to bonding confined to the Au region (Figure S18a, Supporting Information). In contrast, samples prepared with LBPW showed virtually no wrinkles in the overlapping substrate region, attributable to the comprehensive bonding of both the Au electrode and the parylene polymer (Figure S18e, Supporting Information). Furthermore, when subjected to breakage, the stacked bonding sample via WVPAB exhibited stress concentration on the electrode (Figure S18b, Supporting Information), whereas LBPW samples experienced stress uniformly across the entire bonding surface (Figure S18f, Supporting Information). Notably, the bonding force in LBPW was greater than ten times that observed in WVPAB (Figure S18c,g, Supporting Information), highlighting the superior mechanical integrity and reliability of the LBPW bonding method.

Additionally, in the case of WVPAB, wire breakage was precipitated by rapid peeling of the electrode (Figure S18d, Supporting Information). Conversely, in LBPW bonding, each layer was robustly bonded, such that when double‐sided tape was used to secure the sample to the jig, it peeled off before any delamination between the sample layers could occur. This led to the tape tearing the sample, resulting in wire breakage that ceased conductivity but occurred without any layer delamination (Figure S18h, Supporting Information). Therefore, LBPW ensured mechanically and electrically reliable integration, effectively dispersing stress concentration on the electrode parts caused by bending, twisting, stretching, and other mechanical deformations typical in flexible electronics applications.

To evaluate the cyclic durability of samples bonded using LBPW, tests involving 30% compression and repeated bending were conducted. The results demonstrated outstanding mechanical durability, with an 18% increase in resistance (from 5.0 to 5.9 Ω) following 10 000 compression cycles (Figure S19, Supporting Information) and a 3% decrease in resistance (from 4.5 to 4.4 Ω) after repeated bending cycles (Figure 3f). To evaluate the conformability of the bonding region, LBPW‐bonded and ACF tape‐bonded samples were contoured onto a 3D‐printed convex shape. The ACF‐bonded region was unable to deform along a radius smaller than 5 mm due to its thicker bonding layer (Figure S20, Supporting Information). In stark contrast, the LBPW‐bonded region exhibited remarkable flexibility, conforming even along a radius as small as 0.5 mm (Figure 3g; Figure S21, Supporting Information). Consequently, the LBPW method not only enhances mechanical durability but also significantly increases flexibility, benefiting from its hybrid direct‐bonding characteristics.

The electrical characteristics of the LBPW were evaluated by measuring the minimum line and space (L/S) dimensions, minimum bonding area, and contact resistance. Previously, the WVPAB method was constrained by limited bonding area and mechanical bonding strength, as it bonded only the electrodes, achieving an L/S ratio of 10 µm.^[^ ^18^ ^]^ In contrast, the LBPW method demonstrated clear conduction and insulation at a reduced L/S ratio of 5 µm (Figure 3h). Furthermore, due to the hybrid direct bonding capability of LBPW, the samples exhibited comparable fracture strengths across varying L/S values, illustrating robust mechanical properties (Figure S22, Supporting Information). Electrical conduction was verified for a minimum bonding area of 50 µm × 50 µm (Figure S23, Supporting Information). The contact resistance for LBPW was determined by calculating the difference in wiring resistance before and after bonding (Figure S24, Supporting Information), where the wiring included two bonding points, each with a bonding area of 4 mm^2^. The measured difference in wiring resistance was 264 mΩ. Subsequently, the contact resistance for LBPW was calculated to be 0.33 mΩ cm^−^^2^. Therefore, the LBPW method not only enables low‐contact resistance and high‐resolution bonding but also ensures enhanced mechanical durability, highlighting its effectiveness for advanced flexible electronic applications.

### 2.5 Flexible Electronics System via LBPW Bonding Method

**Figure 4:**
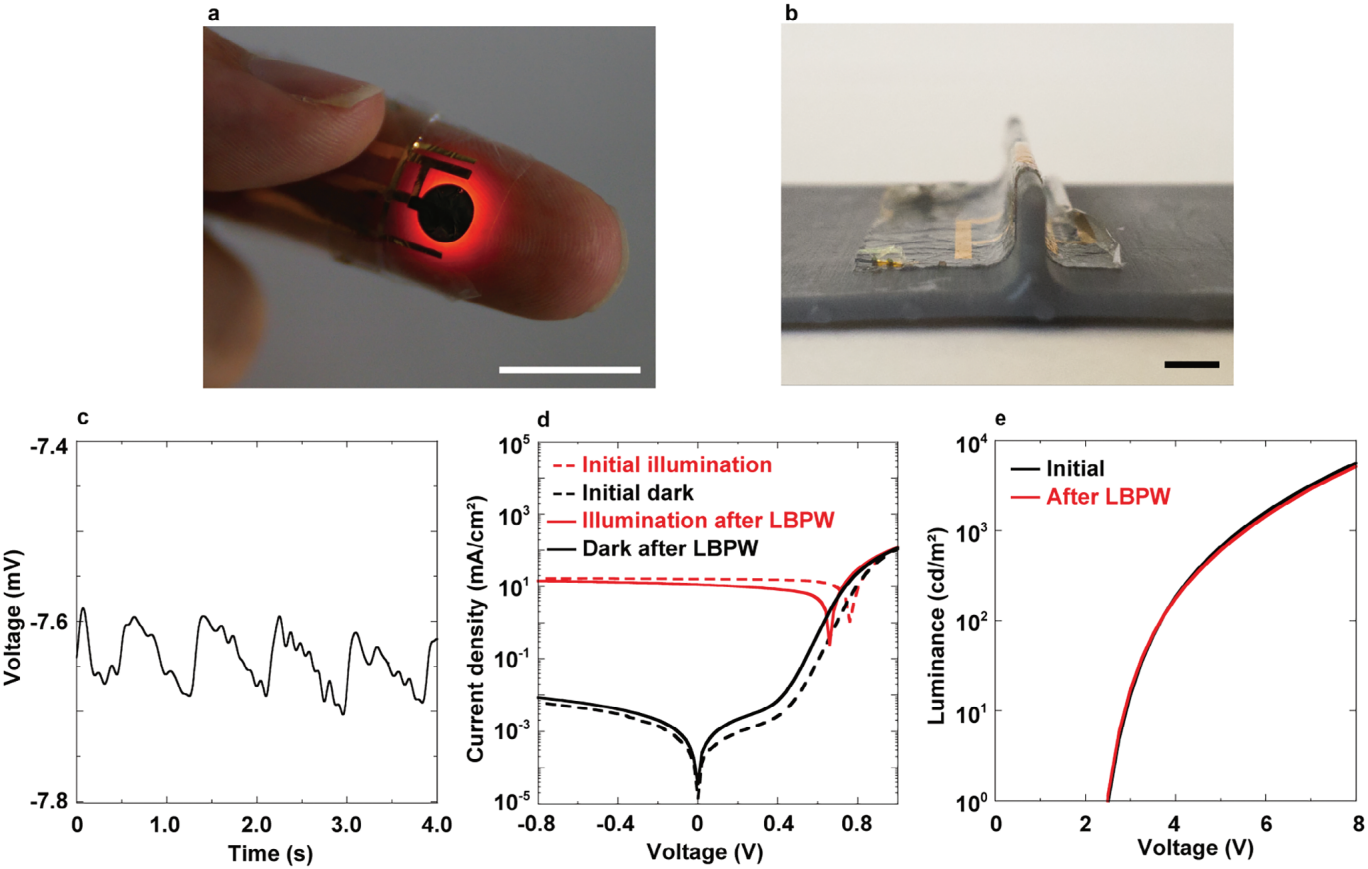
3D‐layer structure of the flexible PPG sensor system using LBPW bonding. a) Image of the stacked structure PPG sensor on a finger. Scale bar: 10 mm. b) Flexibility of the stacked PPG sensor system when applied to a 0.5 mm bump. Scale bar: 2 mm. c) PPG signal detected by the sensor shown in (a). d) Evaluation of OPD performance degradation due to the LBPW process. e) Evaluation of OLED performance degradation due to the LBPW process.

The LBPW method presents a versatile approach for fabricating various structured flexible electronics, thanks to its flexibility, mechanical durability, and high‐resolution bonding capabilities. We demonstrated the effectiveness of this method by constructing a 3D‐stacked multilayer flexible PPG sensor system, integrating an ultra‐flexible OLED and an OPD fabricated on different substrates (**Figure** **4**a; Figure S25, Supporting Information). Utilizing LBPW bonding and double‐sided electrodes via holes on a parylene substrate enabled the successful creation of a 3D‐structured multilayer flexible electronics system. This approach circumvents the complexities associated with 3D fabrication on a single substrate and eliminates the increase in total device thickness typically caused by adhesive layers. The resultant PPG sensor system featured a minimal thickness—4 µm for the OPD and 4 µm for the OLED—with its flexibility derived solely from the thinness of the electronic components (Figure 4b). Additionally, the sensor exhibited robust and reliable bonding between layers due to the comprehensive surface bonding enabled by LBPW. When attached to a fingertip for testing, the sensor reliably produced signals every second, confirming its operational functionality (Figure 4c). Moreover, the degradation of the organic electronics during the LBPW process was minimal for both the OPD (Figure 4d) and OLED (Figure 4e; Figures S26,S27, Supporting Information), ensuring continued effective performance post‐bonding.

LBPW also enables the fabrication of hybrid electronic systems that integrate materials with disparate properties. For instance, it ensures the production of stretchable organic photovoltaics (OPV) by bonding ultra‐flexible OPV, inherently nonstretchable, to stretchable wiring partially coated with evaporated parylene (Figures S28,S29, Supporting Information). This novel combination achieved up to 130% stretchability (with less than 2% degradation), significantly enhancing the nonstretchable ultra‐flexible OPV. The OPV system maintained stable performance, outperforming previous strategies involving prestretched OPV,^[^ ^19^ ^]^ as the device area remained unchanged during stretching (Figure S29e, Supporting Information).

## 3 Discussion

The bonding mechanism of LBPW between the Au electrodes and parylene substrate regions was analyzed separately (Figure S30, Supporting Information). In the case of Au electrodes, the bonding mechanism primarily involves metallic bonds facilitated by atomic diffusion, as evidenced by high‐resolution cross‐sectional observations using TEM and EDX analysis. Given that LBPW is a direct bonding method, achieving complete contact between surfaces is crucial. However, some voids were observed in areas of noncontact, attributed to slight wrinkling caused by manual alignment overlap. To enhance the yield in future applications, the development of jigs that ensure a broader contact area without inducing wrinkling will be crucial.

In the region involving the parylene polymer, the bonding mechanisms are thought to involve fusion bonding^[^ ^20^ ^]^ and partial chemical bonding, primarily due to molecular chain entanglement. Typically, polymer materials exhibit enhanced crystallinity when heated above their glass‐transition temperature (Tg), because such heating enables the mobility and alignment of molecular chains within noncrystalline regions.^[^ ^20^, ^21^ ^]^ Furthermore, intimate contact with similar bulk polymers at temperatures above Tg can eliminate potential energy barriers arising from interfacial inhomogeneities. This effect allows molecular chains to move freely at the interface through interdiffusion. Upon cooling, physical bonding strength emerges due to the entanglement of molecular chains, resulting in robust intermolecular connections.^[^ ^20^, ^21^ ^]^ In the case of parylene polymer bonding via LBPW, the predominant bonding mechanism aligns with this fusion bonding paradigm, as the cross‐sectional analysis reveals the interface to exhibit the highest level of crystallinity.

The untreated parylene polymer exhibits a hydrophobic surface (Figure S31a, Supporting Information) and contains both crystalline and noncrystalline regions (Figure S12, Supporting Information), as is typical for polymers.^[^ ^22^ ^]^ Theoretically, direct bonding of parylene requires heating above its Tg, and previous studies have necessitated thermal compression bonding at temperatures of 160 °C or higher (Figure S1, Supporting Information). Notably, the LBPW method achieves direct bonding at temperatures below 85 °C. This substantial reduction in bonding temperature can be attributed to the functional pretreatment steps integrated into LBPW, which include plasma treatment and water droplet deposition, enhancing the bonding efficiency.

Fusion bonding typically progresses through five continuous stages:^[^ ^20^, ^23^ ^]^ i) surface rearrangement, ii) surface approach, iii) wetting, iv) diffusion, and v) randomization. Each of these stages plays a crucial role in achieving low‐temperature fusion bonding via LBPW. Initially, the parylene substrate undergoes hydrophilic treatment using water vapor plasma, which significantly decreases the water contact angle from 79.1° to 1.5°, despite the inherent hydrophobicity of untreated parylene (Figure S31b, Supporting Information). This significant reduction in water contact angle highlights the effectiveness of the plasma treatment in promoting hydrophilicity through the removal of organic contaminants, oxidation, and molecular chain cleavage from the parylene surface. FTIR measurements further confirm this increase in hydrophilicity, with a notable rise in the peaks associated with OH groups following plasma treatment, indicating enhanced surface hydrophilicity (Figure 2e; Table S3, Supporting Information).

Following plasma treatment, the activated surfaces come into contact, effectively completing the initial steps (i) and (ii) necessary for fusion bonding. The substantial interfacial adhesion achieved meets the requirements of step (iii) for fusion bonding, attributed to the flexible thin‐film characteristic and high surface energy provided by the water‐vapor plasma treatment. Thus, LBPW facilitates effective low‐temperature fusion bonding.

In the LBPW process, water droplets are applied to the hydrophilic surface of parylene. This water penetration into the polymer matrix induces a plasticizing effect, which expands the spacing between polymer chains and reduces intermolecular forces.^[^ ^22^ ^]^ Therefore, water penetrates several nanometers deep from the plasma‐treated parylene surface, reducing the intermolecular forces. Typically, the movement and diffusion of polymer chains within amorphous regions necessitate the disruption of these intermolecular forces. In conventional fusion bonding processes, thermal energy exceeding the Tg is essential to enable polymer chains to move and diffuse freely by breaking these forces. However, DSC measurements (Figure 2d) indicate that while the heated samples exhibited crystallization peaks, the steam‐heated samples—similar to those treated under LBPW conditions—showed no such peaks. This absence of crystallization peaks in steam‐heated samples is expected as these samples had undergone crystallization during earlier heat treatment. This observation suggests that humidity during heating assists in the movement of molecular chains, thereby promoting crystallization and diffusion. The plasticizing effect of water reduces intermolecular forces, ensuring the movement and diffusion of polymer molecules at temperatures below their initial Tg, thereby advancing the diffusion stage (iv). After the low‐temperature heat treatment in the LBPW process, physical bonding forces are presumed to arise from the polymer chain entanglement, completing stage (v) randomization. Plasma treatment with argon or oxygen, in addition to water vapor also decreases the contact angle of parylene, making it hydrophilic (Figure S31c,d, Supporting Information). Consequently, parylene films can be effectively bonded using not only water vapor but also argon and oxygen plasma treatments (Figures S6a,S7a, Supporting Information). The successful bonding in the LBPW process is attributed primarily to physical etching effects such as hydrophilization, surface molecular chain cleavage, and contaminant removal, rather than to chemical alterations induced by plasma treatment.

The lack of bonding in samples treated with water droplets but without plasma treatment is thought to result from inadequate water penetration between the molecular chains and the ineffectiveness of the plasticizing effect (Figures S6a,S7a, Supporting Information). Similarly, samples that did not receive water droplets failed to bond due to the absence of this plasticizing effect (Figures S6c,S7c, Supporting Information).

The direct bonding of polyimide film under the same LBPW conditions used for parylene was also explored. We observed a disparity in bonding effectiveness, which we attribute to the CVD process used for parylene. This process results in lower intermolecular cohesion within the parylene, making it more susceptible to chain scission and water permeation following plasma irradiation. To address these limitations, we propose that the LBPW method can be effectively adapted to bond various materials by carefully optimizing the plasma conditions and heating temperatures, taking into account the initial glass transition temperatures of the respective materials.

## 4 Conclusion 

We developed a direct hybrid bonding method that utilizes parylene and gold electrodes for robust full‐surface bonding. Despite its low processing temperature of 85 °C, the LBPW method can be applied in two significant areas: Au metallic bonding and parylene fusion bonding, without the use of adhesives. The LBPW approach optimizes the use of water vapor plasma, the water‐plasticizer effect, and low‐temperature heating to produce strong surface‐activated bonds for gold and effective low‐temperature fusion bonds for parylene. This study demonstrated that LBPW can robustly integrate flexible electronics both horizontally and vertically. Examples include a stacked structural PPG sensor system and stretchable OPV. Additionally, because the LBPW method does not rely on adhesives, it maintains high flexibility even after integration. With its capability to form metallic bonds in the electrode regions, LBPW achieves excellent high‐resolution integration with a minimum bonding size of less than 50 µm × 50 µm, and it supports resolutions as fine as 5 µm L/S while maintaining mechanical durability. Consequently, LBPW is a practical bonding technology for the fabrication of flexible wearable and implantable devices, offering high‐resolution electrical characteristics and mechanical durability.

## 5 Experimental Section

### Fabrication of the Bonding Ultrathin Film Specimen

Parylene polymer films (dix‐SR, Daisan Kasei Co.) were utilized as ultrathin substrates. Another parylene type, dix‐C (Daisan Kasei Co.), was also employed to assess the dependency of bonding characteristics on the parylene variant. Unless specified otherwise in the figure captions, the thickness of these films was 2 µm. The parylene was deposited onto a supporting glass slide coated with a fluorinated layer (Novec 1700, 3 m) using CVD (PDS2010, Kisco). Patterned Cr (3.5 nm thick) and gold (Au) (100 nm thick) electrodes were deposited on the parylene using a thermal evaporator under a pressure below 1 × 10^−3^ Pa, facilitating simultaneous bonding experiments. The Cr layer served as an adhesion‐promoting interlayer between the parylene and Au.

### LBPW Bonding Procedure

Experiments were conducted with ultrathin film samples fabricated within a 2‐week period. Initially, the ultrathin samples were delaminated from the glass and positioned on the plasma stage for reactive ion etching (RIE) mode in a water vapor plasma machine (Aqua Plasma Cleaner AQ‐500, Samco Inc.) using the following parameters: 50 W plasma power, 12 sccm gas flow, 40 s treatment duration, and 10 Pa pressure. Following plasma treatment, the treated surfaces were exposed to ambient air. To initiate rough Au direct bonding, an overlapping sample was left in ambient air for up to 5 min. DI water was then applied to the overlapped parylene area from the edge using a water contact angle system (DMe‐21, Kyowa Interface Science Co.), enabling precise measurement of the water volume. To ensure uniform contact across the two ultrathin films, including the electrode and substrate areas, the overlapping region was manually pressed with ≈2 N force for 5 s. The samples were then heated in an oven without applying further pressure. An illustration of the LBPW process is shown in Figure S2 (Supporting Information).

### Bonding Alignment

For resolution‐related experiments, the bonding alignment protocol described in ref.[18] was adhered to. For other experiments, alignment was manually performed using tweezers under a microscope.

### Surface STEM and Cross‐Sectional STEM Observation

Specimens for cross‐sectional observation were prepared using an ion beam milling system (EM TIC 3X, Leica) and a microtome (Ultracut‐UCT, Leica). STEM images were captured using an ultra‐versatile high‐resolution SEM (Quattro S, FEI) at an acceleration voltage of 30 kV. In Figure 1b, the black dots near the interface indicate the Au nanoparticles used for focusing.

### Cross‐Sectional TEM Observation

For cross‐sectional observation of the bonded Au region, specimens were prepared with a fast ion‐beam milling system. TEM images were captured using an H9500 microscope (Hitachi High‐Tech Co.) at an acceleration voltage of 300 kV.

### Cross‐Sectional EDX Analysis

Similar preparation methods were used for the bonded Au region intended for EDX analysis. TEM images for this analysis were obtained using an HD2700 (Hitachi High‐Tech Co.) instrument with an acceleration voltage of 200 kV. The analyzed area corresponds to that shown in the expanded TEM image in Figure 1c. A 100 nm EDX line scan was conducted through the gold bonding region and bonding interface, as illustrated in Figure S3 (Supporting Information), with the bonding interface located ≈50 nm from the scanning start point.

### XRD Measurement

Two‐micrometer‐thick parylene films underwent three distinct pretreatments for XRD measurements. The first sample was untreated parylene. The second sample underwent water vapor plasma treatment (12 sccm, 50 W, 40 s, RIE mode). The third sample received the same plasma treatment followed by steam treatment (85 °C, 85%rh, 2 h). To prevent wrinkling, the parylene films were not delaminated from the glass substrate and were measured in the in‐plane mode using a Smart lab system (Rigaku Co.).

### FTIR Measurement

Ten‐micrometer‐thick parylene films, subjected to three distinct pretreatments, were prepared for analysis. The first sample was untreated parylene. The second sample underwent water vapor plasma treatment (12 sccm, 50 W, 40 s, RIE mode). The third sample received the same plasma treatment followed by steam treatment (85 °C, 85%rh, 4 h). To prevent wrinkling, the parylene films were kept on the glass substrate and analyzed using the attenuated total reflection method (IRSpirit, Shimadzu Co.). Figure 2e presents the baseline‐corrected data by the LabSolutions IR (Shimadzu Co.).

### DSC Measurement

Two‐micrometer‐thick parylene films underwent two different pretreatments. The first sample was subjected to water vapor plasma treatment (12 sccm, 50 W, 40 s, RIE mode) followed by steam treatment (85 °C, 85%rh, 21 h). The second sample received the same plasma treatment but was then oven‐heated (85 °C, 21 h). DSC analysis was conducted using a DSC‐60 Plus (Shimadzu Co.), and Figure 2d shows the baseline‐corrected data by the LabSolutions TA (Shimadzu Co.).

### XPS Measurement

A bonded sample with 10‐µm‐thick parylene films prepared using the LBPW method was analyzed. Following a 90° delamination test, the bonded samples were forcibly delaminated. Due to the sample thickness, some regions remained bonded while others did not. Both types of surfaces were analyzed using an XPS photoelectron spectrometer (KRATOS ULTRA2 (AXIS Supra), Shimadzu Co.).

### AFM Measurement

A sample for AFM measurements was prepared by depositing a parylene film on glass. Surfaces of parylene that had undergone water vapor plasma treatment and those left untreated were analyzed using AFM (N9613A, Keysight).

### 90° Delamination Test

Parylene films, 2 µm thick and measuring 10 mm × 24 mm, were prepared. After subjecting them to various plasma irradiation conditions, the two treated parylene surfaces were brought into contact as illustrated in Figure S5a (Supporting Information), with an overlapping area of 10 × 10 mm. DI water was then injected along the edge of the overlapped parylene area under specific research conditions, and the sample was subsequently heated in an oven. Following the LBPW process, double‐sided tape was affixed to one side of the bonded sample on the glass. A PI film was attached to the edge on the opposite side. The sample was then mounted in a tensile testing machine (EZ‐LX, Shimadzu Co.), as shown in Figure S5b (Supporting Information), and immersed in water for 3 min prior to initiating the delamination test. The test proceeded at a speed of 10 mm min^−1^.

### Definition of Bonded or Nonbonded

The term “bonded” refers to cases where the parylene substrate fractures rather than the bonding interface itself (substrate failure mode), during destructive testing. Conversely, “non‐bonded” describes instances where the specimen fractures and delaminates at the bonding interface (either parylene/parylene or Au/Au) indicating an adhesive failure mode, as depicted in Figure S32 (Supporting Information).

### Cyclic Bending Test

Bonded samples prepared using the LBPW method are depicted in Figure S19a (Supporting Information). For the bending test, the sample was laid on a 50‐µm‐thick supporting film to maintain a consistent bending radius of 1.0 mm, which was then secured to a movable stage. The sample was included in a circuit with a three‐terminal regulator functioning as a resistor. The voltage across the sample was recorded for each 1 mm movement of the stage, and the electrical resistance was calculated from these voltage readings. The stage movement spanned a total of 10 mm. The sample number was one sample.

### Evaluation of the 3D‐Stacking Bonding

Au (50 nm) / Cr (3.5 nm) layers were deposited onto a fluorinated‐layer‐coated glass substrate to form the bottom electrode using a thermal evaporator. After depositing 3 g of parylene, via‐holes were created using a green laser on both the parylene and the underlying Au electrodes. Subsequently, Cr (3.5 nm) and Au (100 nm) were deposited to form a 5 mm × 5 mm bonding pad. The parylene samples with double‐sided electrodes were bonded using either LBPW or WVPAB methods. For LBPW, 2.0–3.0 µL of DI water was injected. The remainder of the procedure followed the standard LBPW process. The bonded samples were then affixed to a tensile testing machine (Figure S17, Supporting Information), with the test speed set to 10 mm min^−1^. During the tensile test, changes in the wiring resistance were monitored using a digital multimeter (34465A, Keysight).

### Plasma Gas Comparison for the Parylene Low‐Temperature Direct Bonding Optimization

This study evaluated the dependency of low‐temperature direct bonding of parylene on different plasma gases, including oxygen, argon, and water vapor. For argon and oxygen gas plasma, a plasma treatment machine (PC‐300, Samco Inc.) was employed. Water vapor plasma treatments were conducted using an Aqua Plasma Cleaner AQ‐500 (Samco Inc.). Following the bonding process, the bonding strength was assessed using a 90° delamination test.

### Evaluation of Parylene‐Type Dependency for Bonding

Two‐micrometer‐thick parylene films were directly bonded using water vapor plasma treatment (50 W, 12 sccm, 40 s, RIE mode, 10 Pa), with 0.5 or 1.0 µL of DI water, followed by oven heating at 85 °C for 4 h, consistent with other dix‐SR samples. The bonding strengths of these samples were measured using a 90° delamination test.

### Evaluation of Contact Resistance for LBPW Bonding

Three thin‐film electrode samples were fabricated with a design that minimizes wiring resistance (Figure S24, Supporting Information). The electrical resistance of each sample was accurately measured using the four‐terminal method with a semiconductor device parameter analyzer (B1500a, Keysight). These thin‐film samples were then directly bonded using the LBPW method, as illustrated in Figure S24 (Supporting Information). Each bonded thin‐film sample featured two bonding areas, each measuring 4 mm^2^.

To evaluate the electrical resistance of the entire circuit, measurements were again performed using the four‐terminal method. The contact resistance for the LBPW method was calculated by determining the difference between the sum of the electrode resistances (R_electrode 1_ + R_electrode 2_ + R_electrode 3_) and the resistance of the bonded circuit (R_bonded electrode_). This difference was then divided by the number of bonding points, which, in this case, represented two.

### Fabrication of an Ultra‐Flexible OPD Module

A 1 µm‐thick parylene film (diX‐SR, Daisan Kasei Co.) was deposited onto a glass plate coated with a fluorinated polymer layer (Novec 1700, 3 M) using CVD. An epoxy solution was prepared by mixing epoxy (SU‐8 3005, MicroChem) and solvent (SU‐8 Developer, MicroChem) in a 1:1 weight ratio for 30 s with a vortex mixer, followed by deposition of a 500 nm‐thick SU‐8 solution layer as the planarization layer. The substrates were then annealed in a nitrogen atmosphere at 180 °C for 30 min.

A 100 nm‐thick indium tin oxide (ITO) layer was sputtered without substrate heating and patterned via photolithography and wet etching to serve as the bottom electrode. Cr/Au contact pads (3.5 nm Cr and 100 nm Au) were fabricated on the ITO layer using thermal evaporation at a pressure below 1 × 10^−3^ Pa. Subsequently, a ZnO nanoparticle layer (HTD‐711Z, TAYCA Co.) was applied as the electron‐transporting layer. This layer was spin‐coated at 5000 rpm for 30 s using a precursor solution of ZnO nanoparticles (1 mL) dissolved in 3‐pentanol (10 mL), followed by baking at 180 °C for 30 min after selective patterning with acetone. An active layer solution was prepared by dissolving poly[4,8‐bis(5‐(2‐ethylhexyl) thiophene‐2‐yl) benzo[1,2‐b;4,5‐b']dithiophene‐2,6‐diyl‐alt‐(4‐octyl‐3‐fluorothieno[3,4‐b]thiophene)‐2‐carboxylate‐2,6‐diyl] (PBDTTT‐OFT) (9 mg) and [6,6]‐phenyl‐C_71_‐butyric acid methyl ester (PC_71_ BM) (11 mg) in 980 µL chlorobenzene and 20 µL 1,8‐diiodooctane, stirring for 5 h at 70 °C and 400 rpm. This solution was spin‐coated in a glovebox at 300 rpm for 240 s to produce a 100 nm‐thick photoactive layer, followed by drying for 0.5 h in a dark vacuum chamber.

To complete the device, MoO_X_ (7.5 nm) and Ag (100 nm) layers were sequentially deposited as the hole‐transporting layer and anode, respectively, using thermal evaporation at a pressure below 1 × 10^−3^ Pa. Finally, a 1 µm‐thick parylene layer was applied as a passivation layer using CVD.

### Fabrication of an Ultra‐Flexible OLED

Bottom Au/Cr electrodes (100 nm Au, 3.5 nm Cr) were deposited as bonding pads onto Novec‐coated glass using a thermal evaporator at a pressure below 1 × 10^−3^ Pa. A 2 µm‐thick parylene film (diX‐SR, Daisan Kasei Co.) was then deposited via CVD to form an ultrathin substrate. An epoxy solution was prepared by mixing epoxy (SU‐8 3005, MicroChem) with a solvent (SU‐8 Developer, MicroChem) in a 1:1 weight ratio for 30 s using a vortex mixer. This solution was spin‐coated to create a 500 nm‐thick SU‐8 planarization layer (5000 rpm, 60 s), which was then prebaked at 95 °C for 3 min, followed by UV exposure and post‐baking at 95 °C for an additional 3 min. The substrates were subsequently annealed in a nitrogen atmosphere at 180 °C for 30 min. A 100 nm‐thick layer of indium tin oxide (ITO) was sputtered as the bottom electrode, without substrate heating, and patterned via photolithography and wet etching. Conductive vias were created to connect both sides of the parylene layer by laser cutting through the parylene and SU‐8 layers with a green laser. Cr/Au electrodes (5 nm Cr, 50 nm Au) were then deposited as contact pads over the ITO layer and via holes using thermal evaporation at a pressure below 1 × 10^−3^ Pa.

A hole injection layer (Clevios Al4083, Heraeus Co.) was applied by spin‐coating at 1210 rpm for 40 s, followed by annealing at 120 °C for 10 min in ambient air. The electron transport and emission layers were applied via spincoating, while NaF (1 nm) and Al (100 nm) were deposited as the cathode. A final passivation layer (NPS812, TOYOCHEM Co., Ltd.) was applied by spincoating at 3000 rpm for 30 s and UV‐cured for 250 s in a nitrogen atmosphere.

### Fabrication of an Ultra‐Flexible PPG Sensor

A low‐adhesion tape (≈1 mm × 30 mm) was affixed to the edge of the ultra‐flexible OLED to facilitate delamination from the glass substrate. Both an ultra‐flexible OLED (now free‐standing) and an ultra‐flexible OPD (prior to delamination) were plasma‐treated with water vapor gas (50 W, 12 sccm, 40 s, RIE mode). The OLED was then aligned on top of the OPD with bonding pads manually adjusted. Subsequently, 1.2 µL of DI water was introduced at the edge of the overlapped parylene region. This overlapped assembly was then heated in an oven under a nitrogen atmosphere (DT300H, Yamato Scientific Co., 10 L min^−1^ flow rate). To prepare external wiring, a layer of Cr/Au (3.5 nm Cr, 100 nm Au) was deposited on a 12.5‐µm‐thick polyethylene naphthalate film via thermal evaporation. Following the heating process, this external wire was attached to the PPG sensor, which was then carefully delaminated from the glass substrate.

### PPG Measurement

The ultra‐flexible PPG sensor was affixed to a finger using a 6‐µm‐thick transparent double‐sided tape, and the PPG signal was detected with a measurement system supplied by Konica Minolta, Inc. The processed signal, shown in Figure 4c, was filtered using the Fast Fourier Transform (FFT) and Inverse Fast Fourier Transform (IFFT) methods with a cutoff frequency of 10 Hz. All experiments were approved by the Ethics Committee of the University of Tokyo (approval no. KE23‐65).

### Degradation Analysis of the LBPW Process Using OPD and OLED

To assess potential degradation from the LBPW process, OPD and OLED devices were fabricated on glass substrates using the same structures and materials as the ultra‐flexible OPD and OLED. Initial current density–voltage (*J–V)* characteristics of the OPDs were measured under AM 1.5G illumination (100 mW cm^−2^, calibrated with a silicon reference cell) using a Keithley 2400 source meter in ambient conditions. Each OPD was then plasma treated with water vapor gas (50 W, 12 sccm, 40 s, RIE mode), and a 1.0 µL water droplet was applied to the edge of the device. After heating in a nitrogen atmosphere (85 °C, 2 h), OPD performance was reevaluated. The OLED devices were similarly evaluated, as shown in Figure 4e and Figure S25 (Supporting Information). Following initial measurements, one OLED device underwent plasma treatment with water vapor gas (50 W, 12 sccm, 40 s, RIE mode), with 2.0 µL water droplets applied at the electrode edge. After subsequent heating in a nitrogen atmosphere (85 °C, 2 h), performance measurements were repeated. Additionally, control OLED devices were left in ambient air for 2 h, after which their performance was reevaluated. Changes in the active area were observed and recorded (Figure S27, Supporting Information).

### Fabrication and Measurement of the Stretchable OPV

To fabricate the stretchable OPV, Au/Cr electrodes (100 nm Au, 3.5 nm Cr) were first deposited as bonding pads on a Novec‐coated glass substrate using thermal evaporation at a pressure below 1 × 10^−3^ Pa. A 1 µm‐thick parylene film (diX‐SR, Daisan Kasei Co.) was then deposited onto this substrate via CVD, followed by spin‐coating of a 500 nm‐thick SU‐8 layer to serve as a planarization layer. For the bottom electrode, a 100 nm‐thick indium tin oxide (ITO) layer was sputtered without substrate heating and patterned by photolithography and wet etching. Conductive holes were created through the parylene layer using a green laser. Additional Cr/Au contact pads (3.5 nm Cr, 100 nm Au) were deposited on the ITO layer using thermal evaporation at a pressure below 1 × 10^−3^ Pa.

Subsequently, a ZnO layer was applied as the electron‐transporting layer. This layer was prepared by dissolving a ZnO nanoparticle solution (1 mL) in 3‐pentanol (10 mL), spin‐coated at 5000 rpm for 30 s, and then baked at 180 °C for 30 min in ambient air. For the active layer, 10 mg of poly[4,8‐bis(5‐(2‐ethylhexyl) thiophen‐2‐yl) benzo[1,2‐b;4,5‐b’]dithiophene‐2,6‐diyl‐alt‐(4‐octyl‐3‐fluorothieno[3,4‐b]thiophene)‐2‐carboxylate‐2,6‐diyl] (PBDTTT‐OFT) and 15 mg of 2,2′‐((2Z,2′Z)‐(((4,4,9,9‐tetrakis(4‐hexylphenyl)‐4,9‐dihydro‐sindaceno[1,2‐b:5,6‐b']dithiophene‐2,7‐diyl)bis(4‐((2‐ethylhexyl)oxy)thiophene‐5,2‐diyl)) bis(methanylylidene))bis(5,6‐difluoro‐3‐oxo‐2,3‐dihydro‐1H‐indene‐2,1‐diylidene))dimalononitrile (IEICO‐4F) were dissolved in 970 µL of chlorobenzene and 30 µL of 1‐chloronaphthalene. This solution was stirred for 3 h at 70 °C and 400 rpm, then spin‐coated at 1400 rpm for 60 s in a glovebox to produce a 100 nm‐thick photoactive layer, which was dried for 30 min in a dark vacuum chamber.

Sequential deposition of 7.5 nm MoO_X_ and 100 nm Ag was performed to complete the device, followed by a 1 µm‐thick parylene passivation layer applied via CVD. For the stretchable wiring, a 2 µm‐thick parylene layer was evaporated onto the edge of a 100 µm‐thick stretchable film (Mitsubishi Chemical Co.), and Cr (3.5 µm) / Au (100 µm) was deposited onto this parylene region as a bonding area. Subsequently, EGaIn was spray‐coated over part of the Au bonding pad and the stretchable wire region. The ultra‐flexible OPV was bonded to the stretchable wiring using the LBPW method (Figures S28, S29, Supporting Information). To measure the *J–V* characteristics during stretching, the stretchable wire region of the OPV was secured to a linear stage with double‐sided tape. *J–V* measurements were conducted using a Keithley 2400 source meter under illumination from a solar simulator (100 mW cm^−2^).

### Statistical Analysis

All data without specific mention were analyzed with Excel (Microsoft 365, Microsoft) and Kaleidagraph (version 4.5, Synergy Software).

## Conflict of Interest

The authors declare no conflict of interest.

## Author Contributions

M.T. performed conceptualization. M.T., D.I., K.F., T.Y., and T.S. performed the methodology. M.T., D.I., and L.S. performed investigation. M.T., D.I., K.F., T.Y., M.Y., T.I., and T.S. performed analysis. K.F., T.Y., and T.S. performed supervision. M.T., S.U., K.F., T.Y., M.Y., and T.S. performed writing.

## Supporting information



Supporting Information

Supplemental Movie 1

Supplemental Movie 2

## Data Availability

The data that support the findings of this study are available from the corresponding author upon reasonable request.
