# Supplementary material for: Robust Full‐Surface Bonding of Substrate and Electrode for Ultra‐Flexible Sensor Integration
Source: Adv Mater. 2025 Feb 18;37(49):2417590. doi: 10.1002/adma.202417590 (PMC12691895; doi:10.1002/adma.202417590)
Supplement: Supplementary file 1 — Supporting Information [file ADMA-37-2417590-s002.pdf]

ADVANCED MATERIALS

Supporting Information

for *Adv. Mater.*, DOI 10.1002/adma.202417590

# Robust Full-Surface Bonding of Substrate and Electrode for Ultra-Flexible Sensor Integration

*Masahito Takakuwa, Daishi Inoue, Lulu Sun, Michitaka Yamamoto, Shinjiro Umezu, Daisuke Hashizume, Toshihiro Itoh, Kenjiro Fukuda*, Takao Someya* and Tomoyuki Yokota**

# Supporting Information

**Title**

**Robust Full-surface Bonding of Substrate and Electrode for Ultra-Flexible Sensor Integration**

Masahito Takakuwa, Daishi Inoue, Lulu Sun, Michitaka Yamamoto, Shinjiro Umezu, Daisuke Hashizume, Toshihiro Itoh, Kenjiro Fukuda*, Takao Someya* and Tomoyuki Yokota*

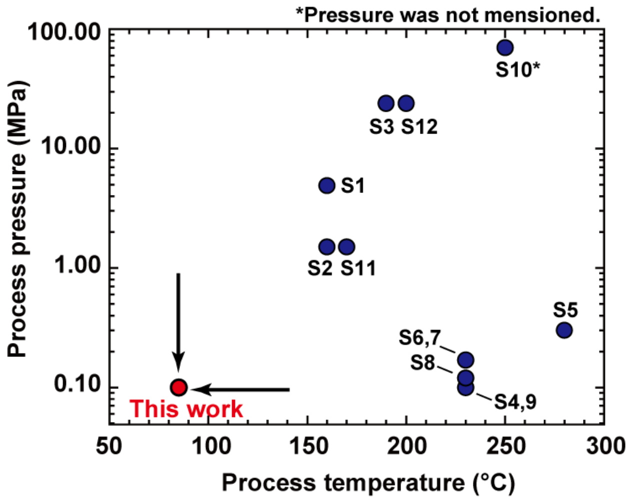


Figure S1 Comparison of the process temperature and pressure for the parylene direct bonding between this study (LBPW) and the previous studies.

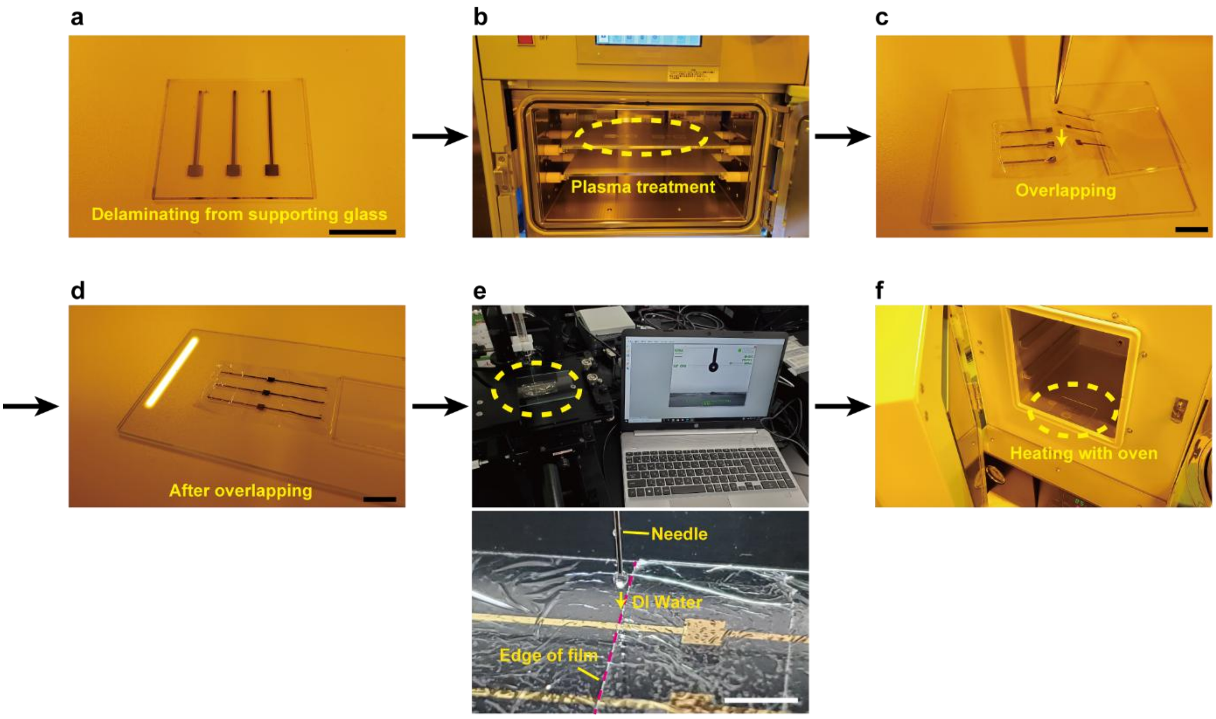


Figure S2 Images of the LBPW procedure.

(a) Parylene polymer substrate with an evaporated Au electrode on a supporting glass. The thin-film sample was delaminated from the glass before plasma treatment. (b) Plasma treatment applied to the bonding surface. (c) After plasma treatment, the activated surfaces were aligned and overlapped using tweezers or a mask alignment system. (d) Overlapped sample positioned on a supporting substrate. (e) Injection of a specified volume of DI water at the edge of the overlapped sample using a contact measurement system. (f) Heating of overlapped samples in an oven. All scale bars represent 10 mm.

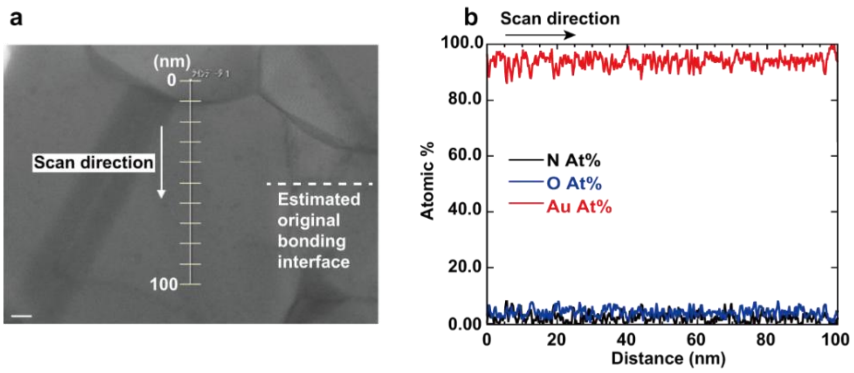


Figure S3 EDX line analysis of the Au direct bonding interface.

(a) Location of EDX line analysis, with a 100 nm line scan. The bonding interface is expected near 50 nm. Scale bar: 10 nm. (b) EDX line analysis results. The bonding interface is expected to be approximately 50 nm.

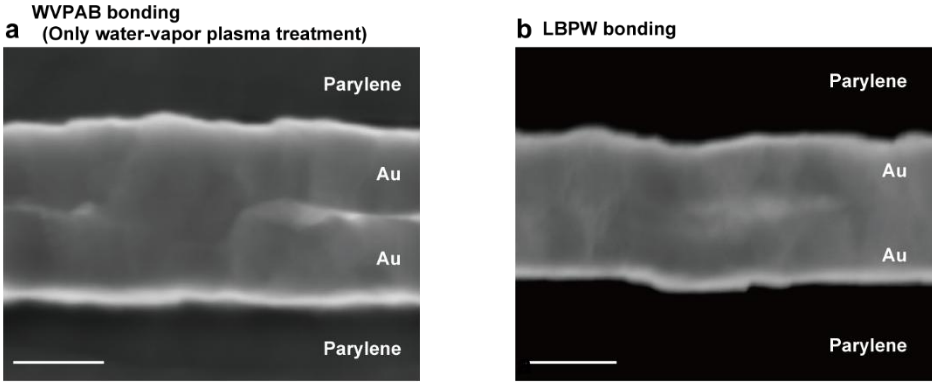


Figure S4 Enhanced Au metal bonding with heating.

(a) Cross-sectional image of Au metal bonding using only water-vapor plasma treatment. (b) Cross-sectional image of Au metal bonding achieved with both water-vapor plasma and heat treatment (100 °C for 3 h). Compared to (a), the Au interface is no longer visible, indicating stronger metallic bonding. All scale bars represent 100 nm.

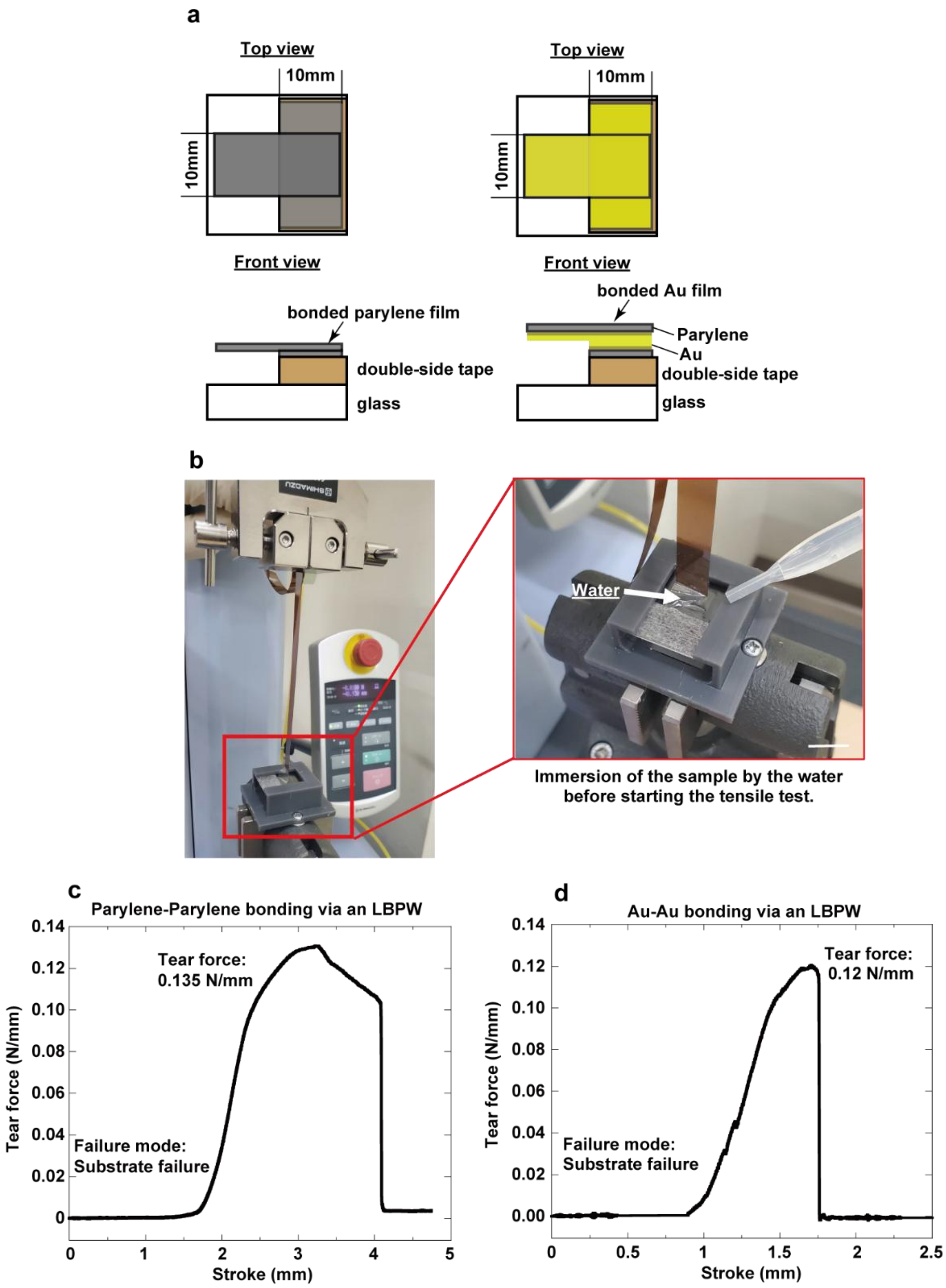


Figure S5 Experimental setup and results of the 90° delamination test.

(a) Thin films were bonded in a T-shape, with a bonding area of 10 mm × 10 mm. After bonding, the sample was affixed to glass with double-sided tape, and a PI film was attached to the opposite edge for clamping. The left design illustrates parylene–parylene bonding, while the right design illustrates Au-Au bonding. (b) Sample fixed to the base of the tensile test machine. Prior to the test, the bonded sample was immersed in water to prevent direct bonding interference from hydrogen in parylene. Scale bar: 10 mm. (c) Results of the delamination test for parylene–parylene bonding. (d) Results of the delamination test for Au-Au bonding.

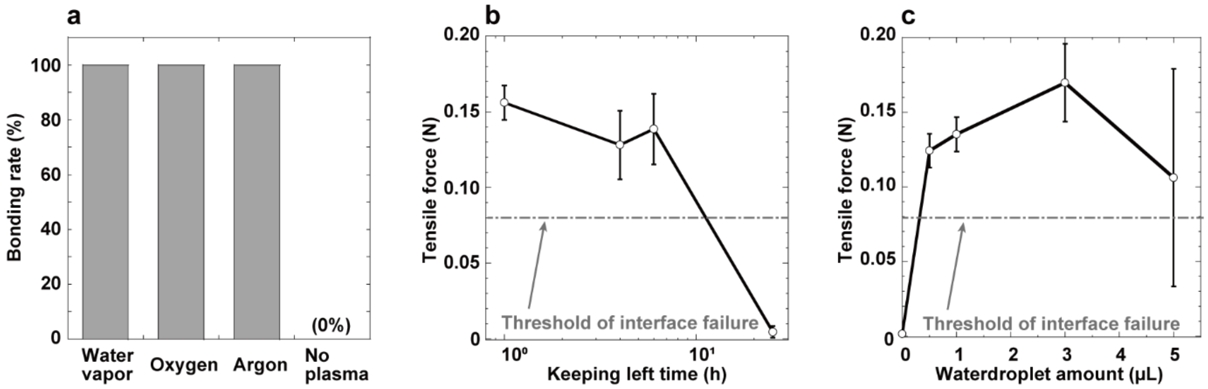


Figure S6. Parylene bonding conditions under various parameters.

(a) Effect of different plasma gas sources on the bonding rate of parylene thin films. Results show samples treated with plasma, followed by the application of a water droplet and heating at 85 °C for 4 h. (b, c) Bonding outcomes under different processing conditions. A tensile strength of >0.08 N/mm indicates substrate failure, while <0.08 N/mm signifies interface delamination. (b) Direct bonding results based on varied waiting times post-plasma treatment. Plasma gas was water vapor, with 1.0 µL water applied. Thermal treatment was conducted at 85 °C for 4 h (mean ± SD, *n* = over 3). (c) Direct bonding results based on different water droplet volumes. Plasma gas was water vapor, with thermal treatment at 85 °C for 4 h (mean ± SD, *n* = over 4).

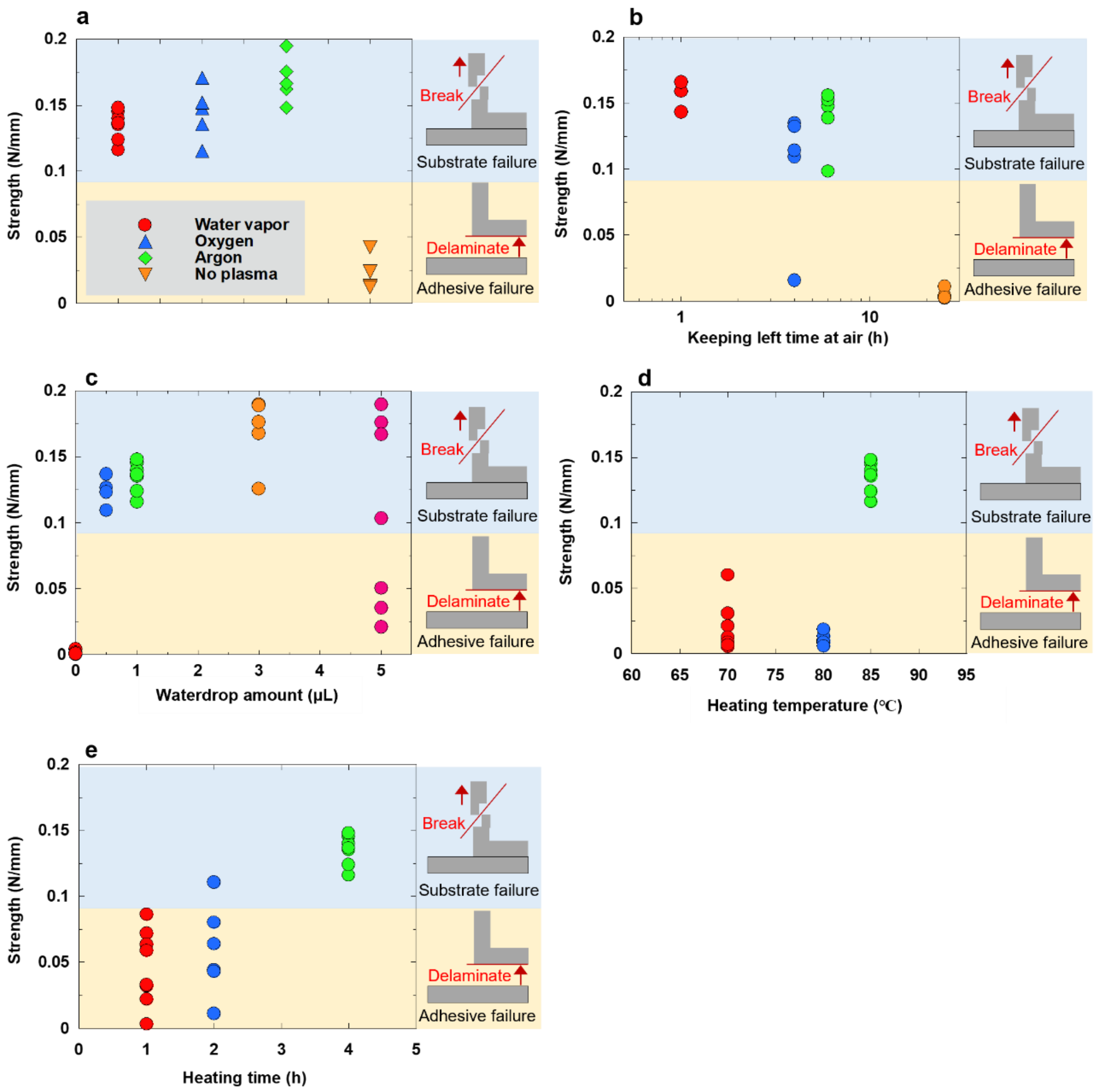


Figure S7. Scatter plots of parylene direct bonding delamination tests under varying conditions. Bond strength >0.08 N/mm indicates substrate failure.

(a) Bonding outcomes based on different plasma gas sources with 1.0 μL of water. Heated at 85 °C for 4 h. (b) Bonding results with varied post-plasma waiting times. Plasma gas was water vapor, with 1.0 μL of water applied. Heated at 85 °C for 4 h. (c) Bonding outcomes based on varying water droplet volumes. Plasma gas was water vapor, with thermal treatment at 85 ℃ for 4 h. (d) Bonding results under different heating temperatures. Plasma gas was water vapor, with 1.0 μL of water. Heating time was 4 h. (e) Bonding results across different heating durations. Plasma gas was water vapor, with 1.0 μL of water and heated at 85 °C.

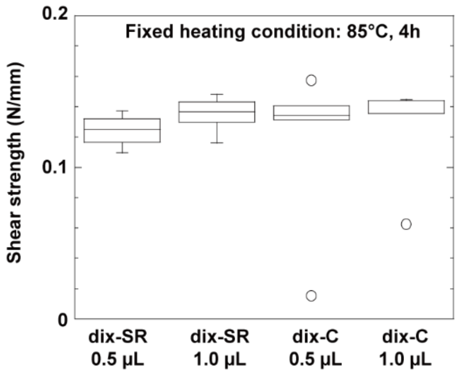


Figure S8. Dependency of parylene type on bonding effectiveness using LBPW. While dix-SR parylene was the primary material in this study, bonding effectiveness of dix-C parylene was also evaluated under LBPW conditions (50 W, 40 s, 12 sccm water-vapor plasma, 0.5 or 1.0 µL DI water, 85 °C for 4 h). A shear force >0.08 N/mm indicates successful bonding (substrate failure) (mean ± SD, *n* = over 4).

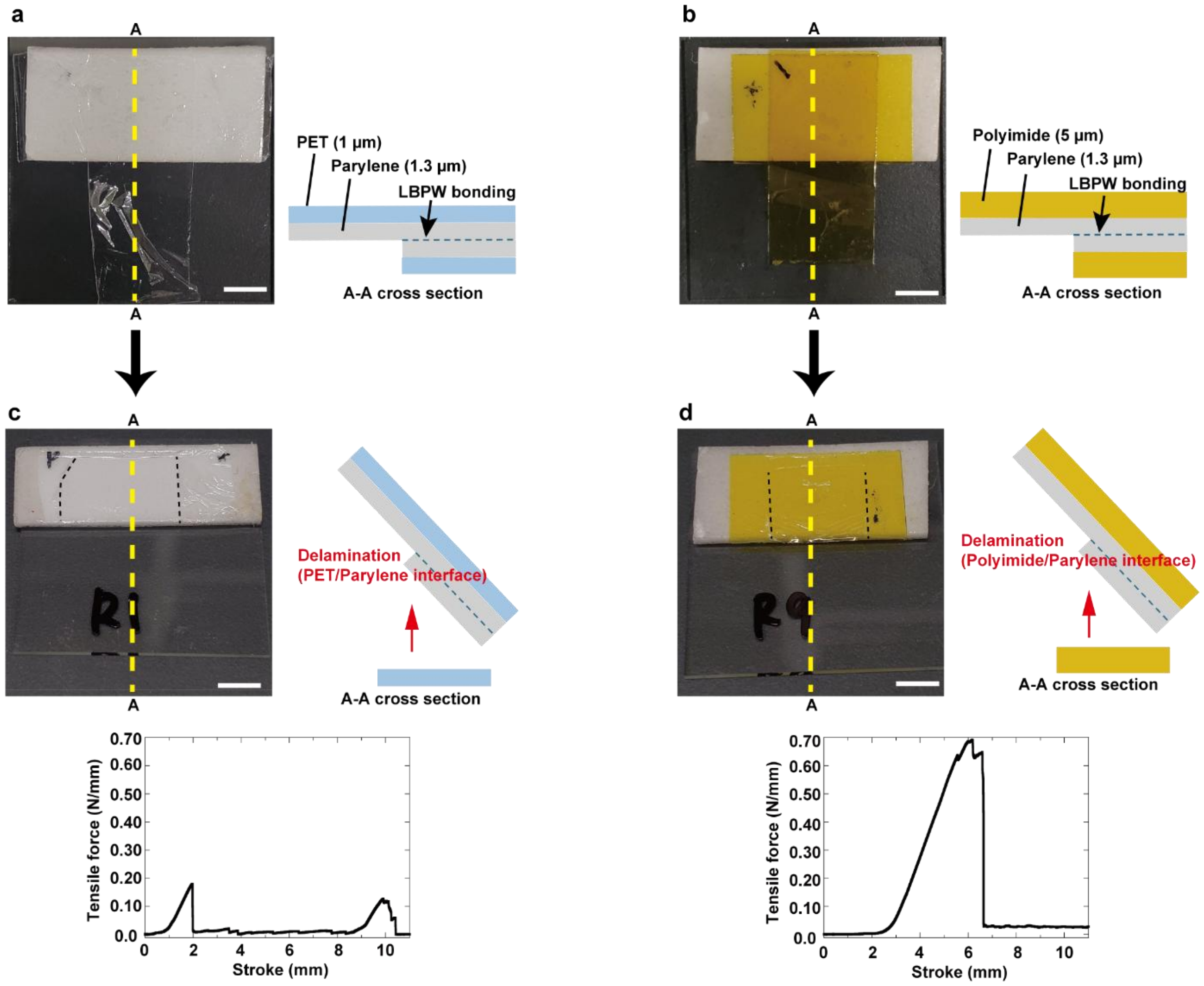


Figure S9. 90° delamination test of bonded polymer films .

(a) Photograph and schematic of bonded PET film covered with parylene. (b) Photograph and schematic of bonded polyimide film covered with parylene. (c) Delamination test results for parylene-covered PET film. (d) Delamination test results for parylene-covered polyimide film. All scale bars represent 10 mm.

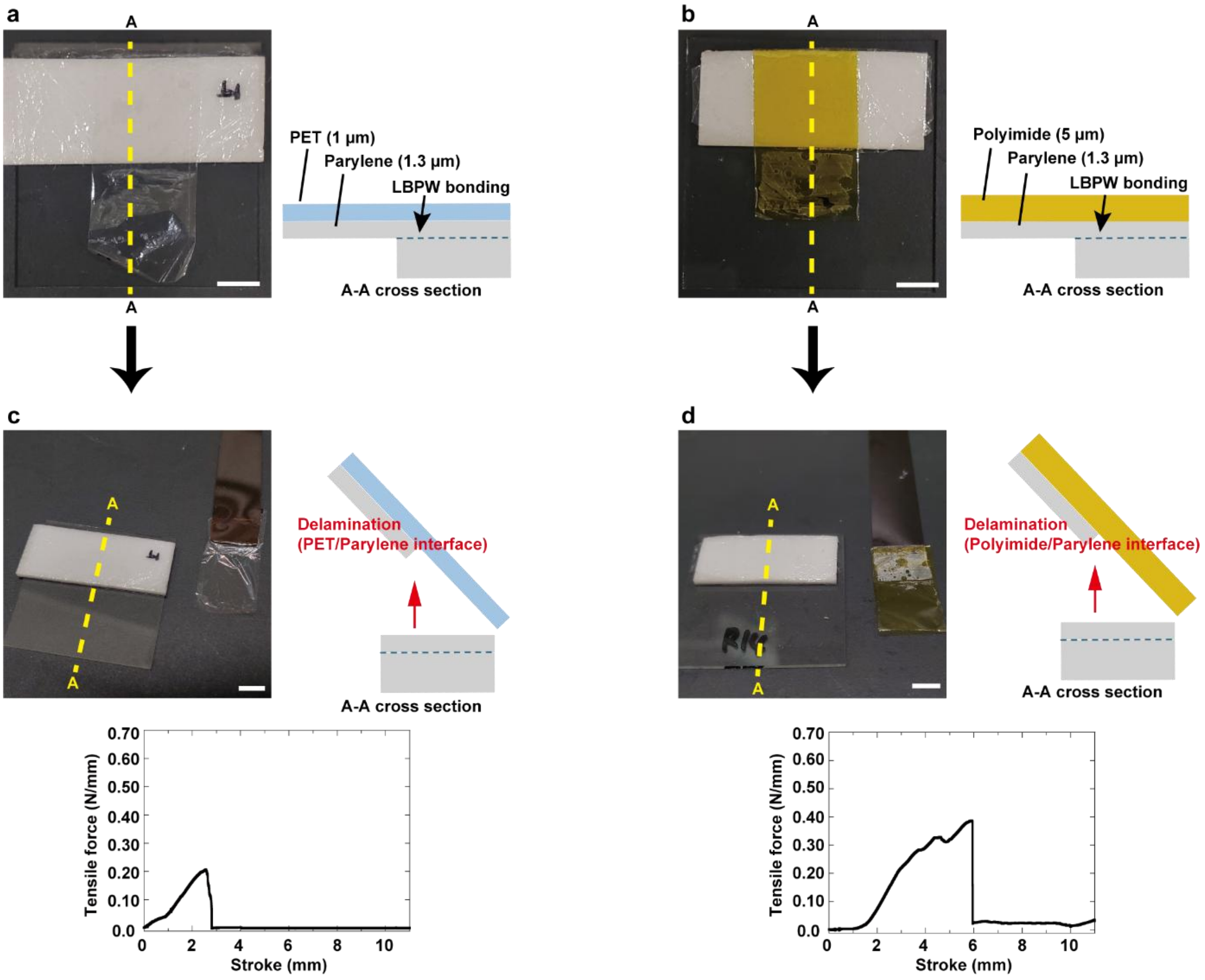


Figure S10. 90° delamination test for bonded parylene and polymer films.

(a) Photograph and schematic of bonded parylene and parylene-covered PET film. (b) Photograph and schematic of bonded parylene and parylene-covered polyimide film. (c) Delamination test results for bonded parylene and parylene-covered PET film. (d) Delamination test results for bonded parylene and parylene-covered polyimide film. All scale bars represent 10 mm.

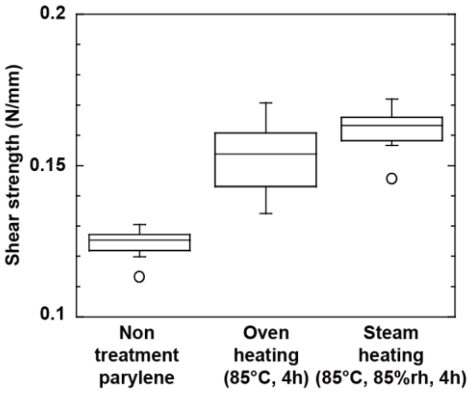


Figure S11. Tensile test results for 2-μm-thick parylene films.

Three types of parylene films were prepared: untreated, water vapor plasma-treated with oven heating, and water vapor plasma-treated with steam heating. Each group included a minimum of eight samples (mean ± SD, *n* = over 8).

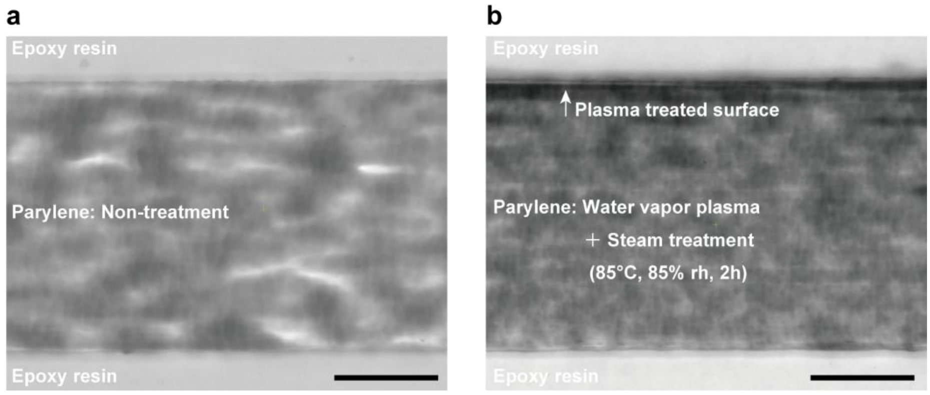


Figure S12. Cross-sectional images of parylene single films.

(a, b) Epoxy resin was used to mount the samples for cross-sectional imaging. Images were obtained in bright-field STEM mode. Lighter regions indicate low electron density and crystallinity, while darker regions represent high electron density and crystallinity. (a) Untreated parylene. (b) Plasma and steam-treated parylene. All scale bars represent 100 nm.

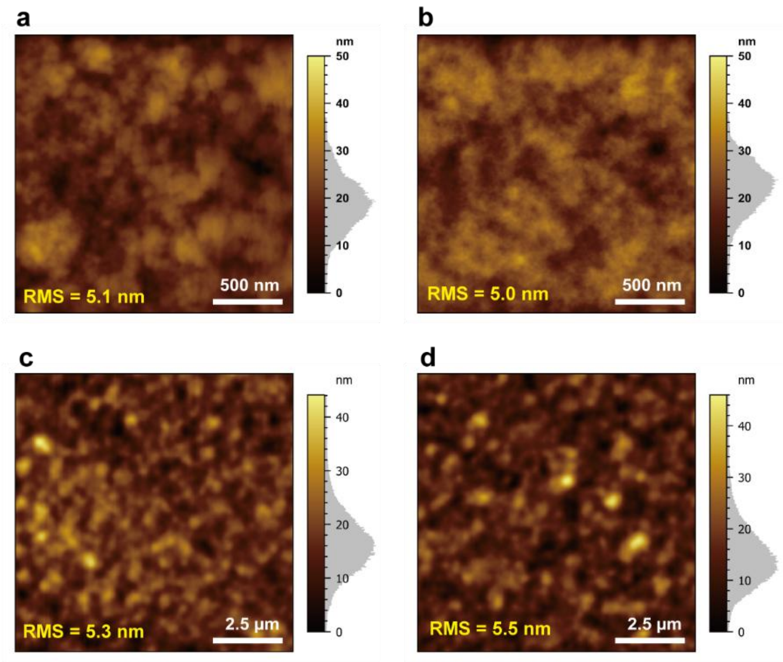


Figure S13. Surface roughness changes in parylene and gold before and after water vapor plasma treatment (50 W, 40 s, 12 sccm, RIE mode). (a) Surface of parylene before plasma treatment. (b) Surface of parylene after plasma treatment. (c) Surface of gold before plasma treatment. (d) Surface of gold after plasma treatment.

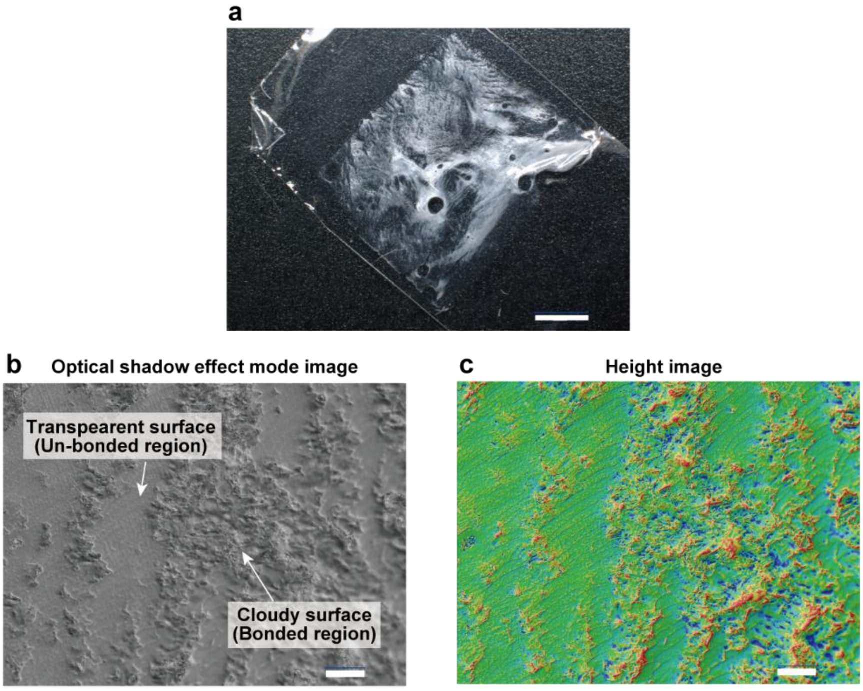


Figure S14. Delamination of parylene surface for XPS analysis.

A 10 μm-thick parylene surface was forcibly delaminated after LBPW bonding.

(a) Optical image. Scale bar: 2 mm. (b) Extended optical shadow mode image. Scale bar: 20 μm. (c) Height image. Scale bar: 20 μm.

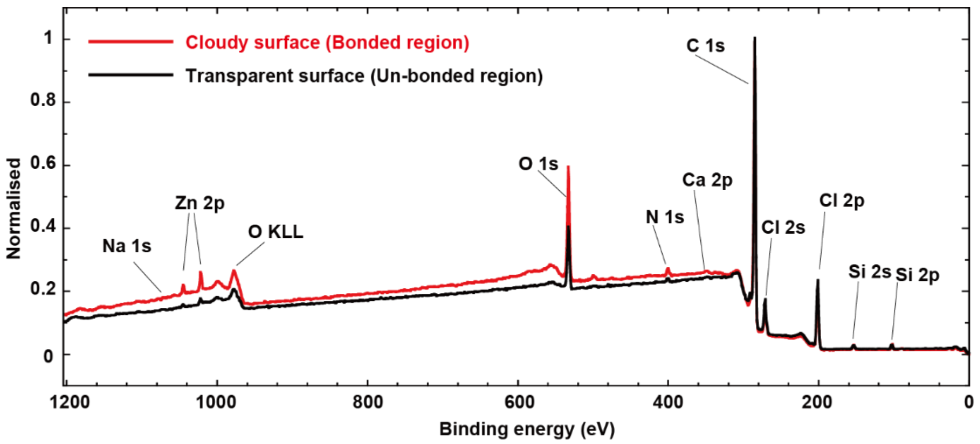


Figure S15. XPS wide spectrum of bonded and unbonded parylene surfaces.

* Traces of N, Na, Zn, Ca, and Si were detected, likely due to residual matter in the equipment. These elements are not expected to influence LBPW bonding directly.

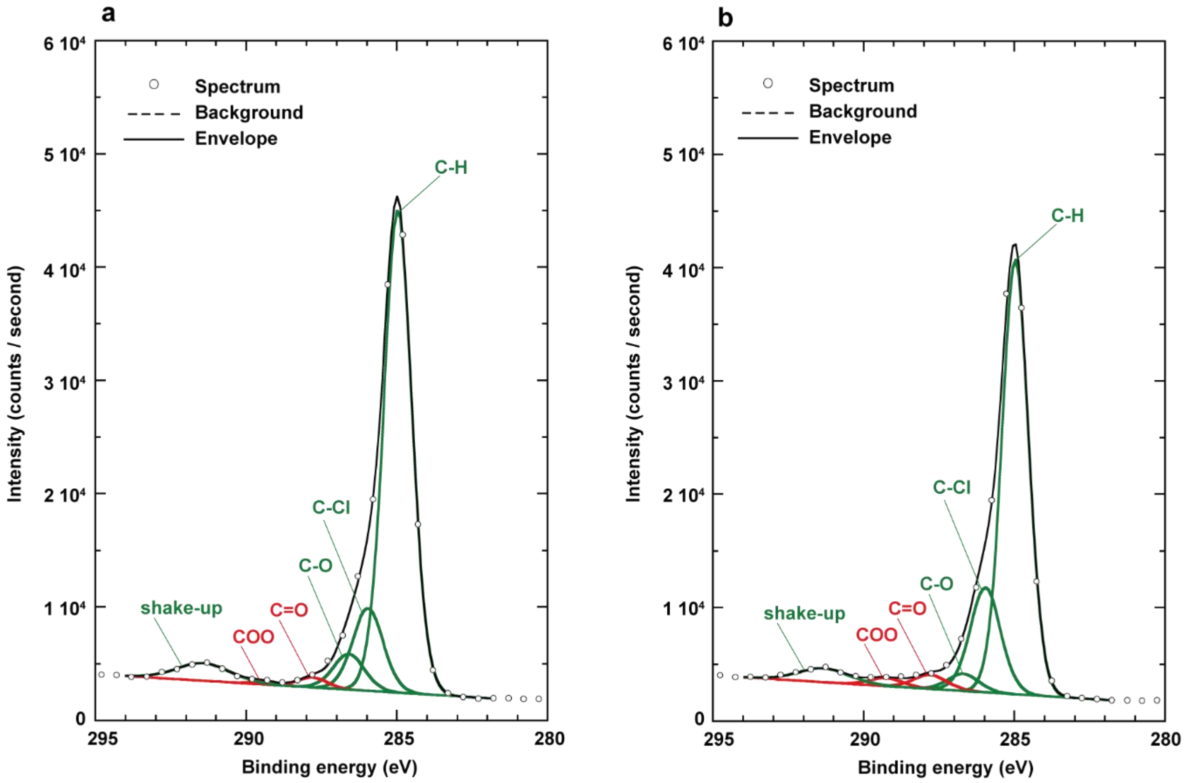


Figure S16. C1s curve fitting spectrum of parylene surfaces obtained via XPS.

(a) Unbonded surface, as shown in Figure S14b. (b) Bonded surface, as shown in Figure S14b.

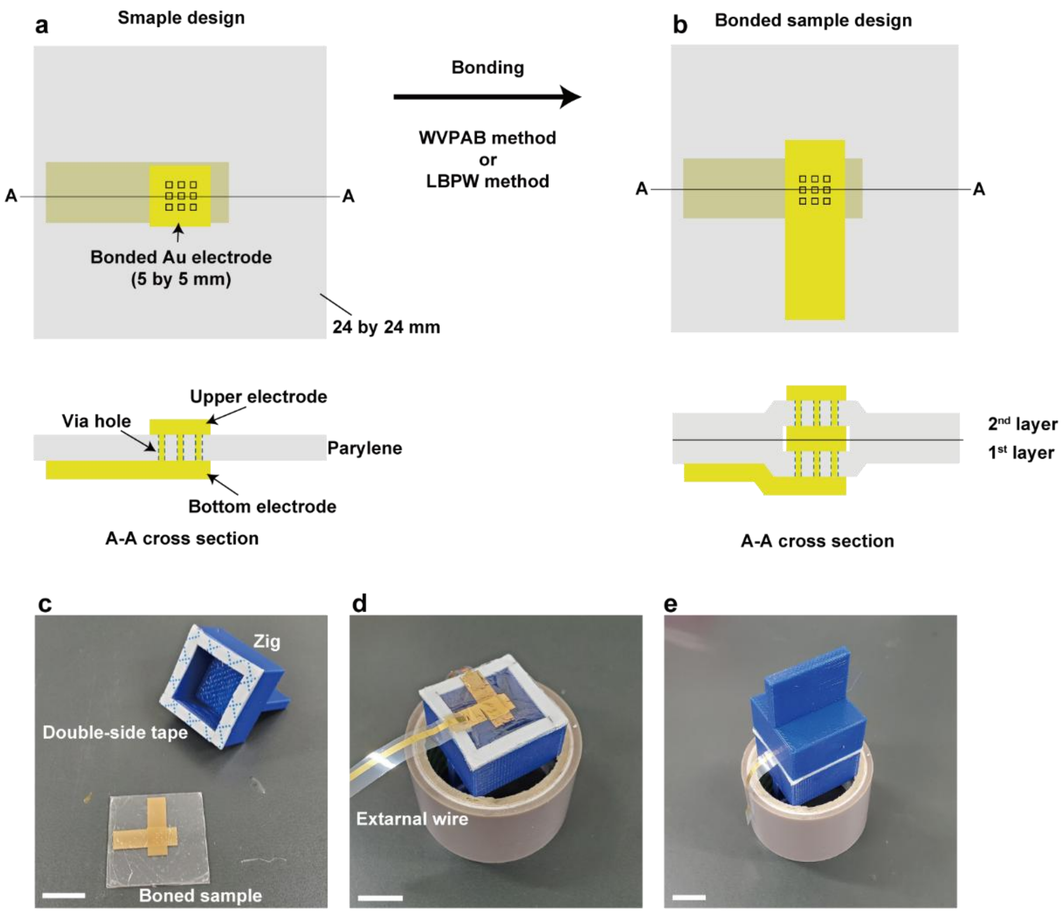


Figure S17. Experimental setup for evaluating mechanical durability between WVPAB and LBPW.

(a) Schematic of sample design, showing double-sided electrodes with via holes. The bottom Au electrode connects to an external electrical measurement system, while the top Au electrode serves as the bonding interface to the second layer's electrode. (b) Schematic of the bonded sample. (c-e) Preparation of the sample for attachment to the tensile testing machine. (c) An external wire adheres to the bottom electrode of the bonded sample using ACF, and one side is secured to the jig with double-sided tape. (d) An external wire adheres to the bottom electrode of the second layer with ACF. (e) The bonded sample of the second layer is affixed to the jig with double-sided tape. All scale bars represent 10 mm.

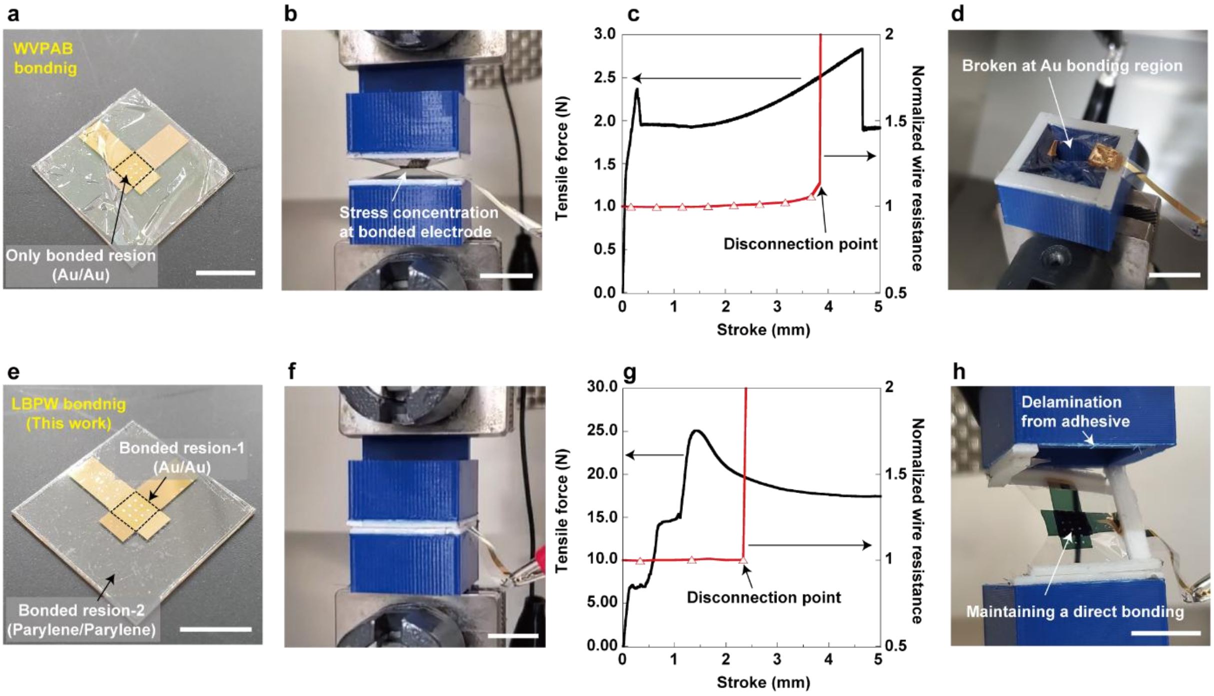


Figure S18. Mechanical durability comparison between WVPAB and LBPW methods via tensile testing.

(a) Image of a sample bonded using the WVPAB method, showing wrinkles in the overlapped substrate region, as WVPAB bonds only the Au region. (b) Image of the sample at the point of electrical disconnection. (c) Tensile test results, showing changes in wiring resistance during testing. (d) Image of the sample after failure. (e) Image of a sample bonded using the LBPW method, with minimal wrinkles in the overlapped region due to full-surface bonding of the Au electrode and parylene polymer. (f) Image of the sample at the point of electrical disconnection. (g) Results of tensile testing and changes in wiring resistance. (h) Image of the sample after failure. All scale bars represent 10 mm.

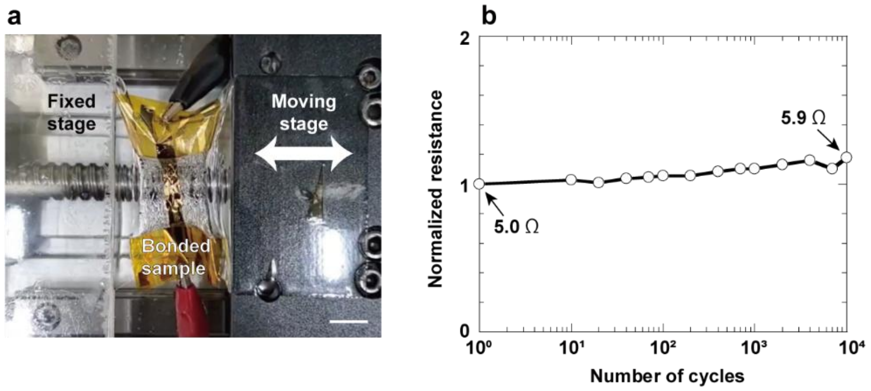


Figure S19. Cyclic compression test.

(a) Measurement setup: the bonded sample was mounted on pre-stretched tape and connected to an external wire for monitoring resistance changes. Scale bar: 10 mm. (b) Resistance change of the bonded sample during compression tests (< 30% compression) over 10,000 cycles.

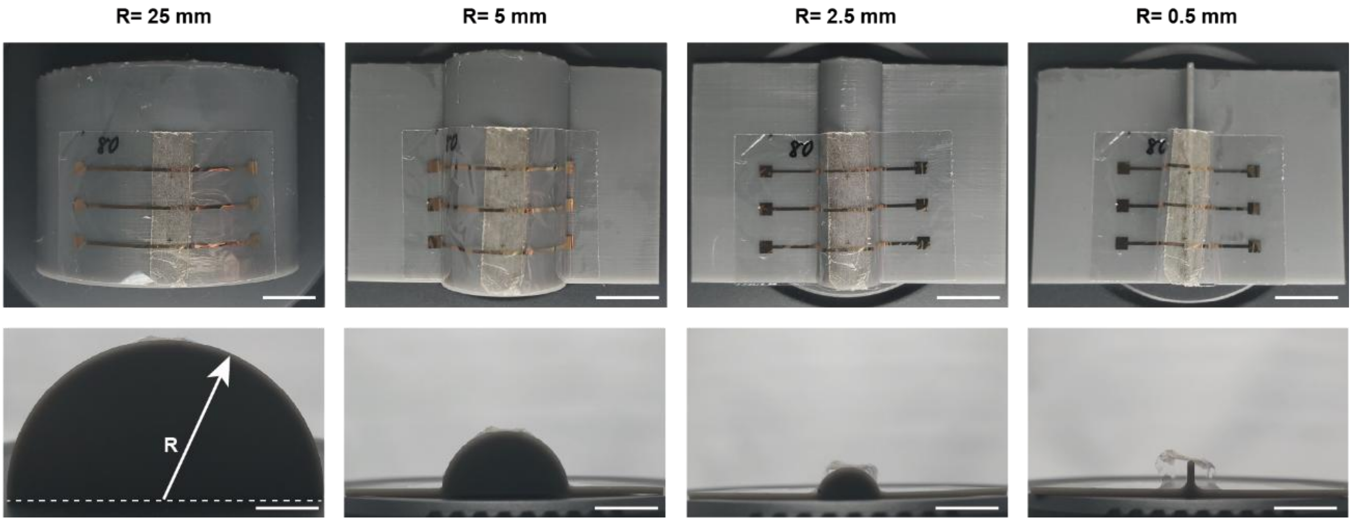


Figure S20. Conformability of the bonding region with ACF tape.

The sample bonded with ACF tape was positioned over a convex surface of various radii (R). The minimum bending radius achieved was < 2.5 mm. All scale bars represent 5 mm.

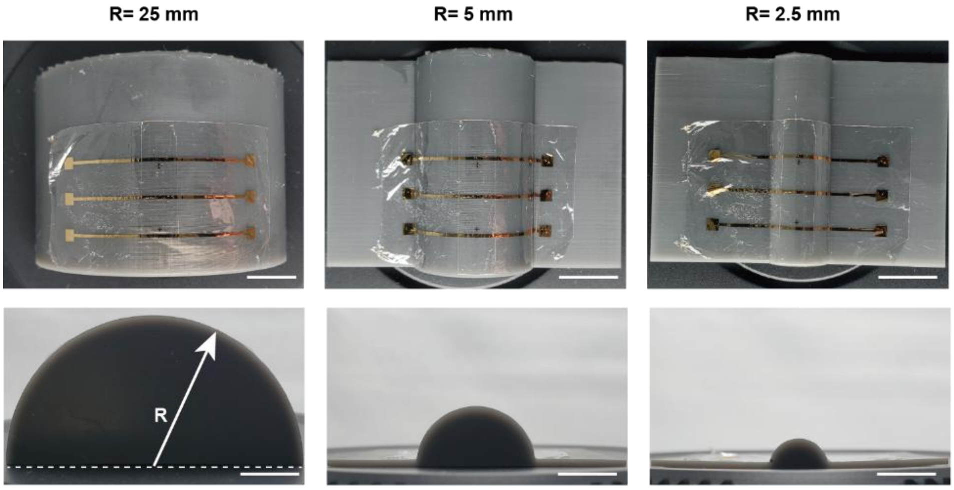


Figure S21. Conformability of the bonding region with LBPW bonding.

Samples bonded via LBPW direct bonding were placed over a convex surface with different radii (R). All scale bars represent 5 mm.

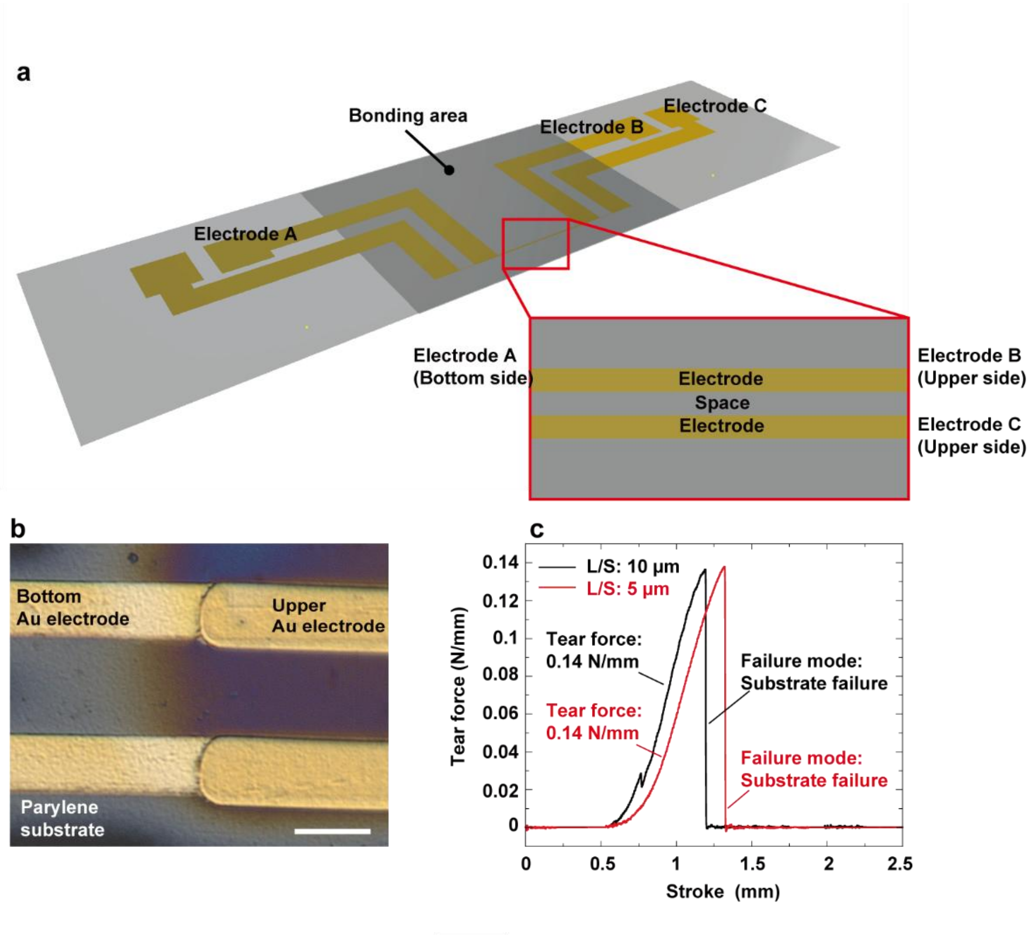


Figure S22. Resolution of the LBPW bonding.

(a) Sample design for evaluating the minimum line/space (L/S) resolution. (b) Optical microscope image of the bonded sample with an L/S of 10 μm. Scale bar: 10 μm. (c) Tensile test results for bonded samples with 5 μm and 10 μm L/S.

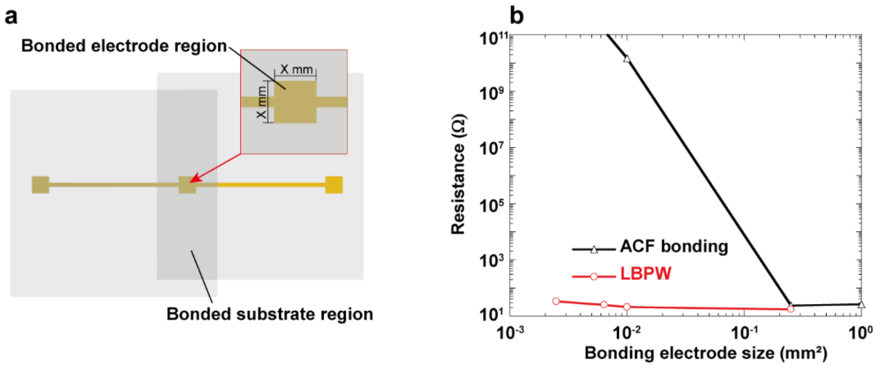


Figure S23. Minimum conductive area in LBPW bonding.

(a) Schematic of sample preparation, where conductivity was evaluated by varying the electrode length, X, between two thin films. (b) Resistance measurements of bonded samples with varying electrode sizes. The black line indicates resistance for samples bonded with ACF tape, while the red line shows resistance for samples bonded via LBPW.

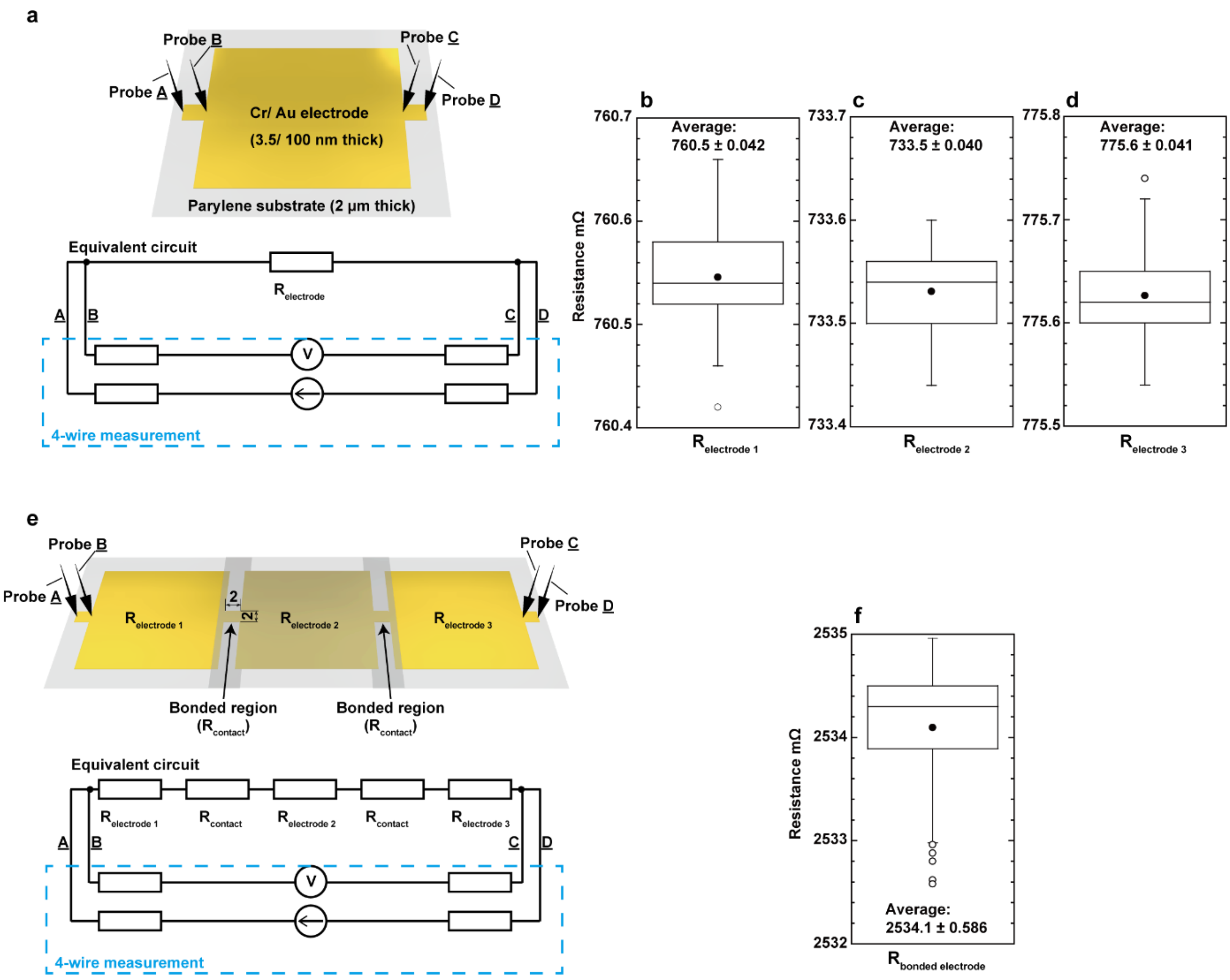


Figure S24. Contact resistance evaluation of LBPW bonding.

(a)Schematic of a single electrode design and its equivalent circuit. (b-d) Electrode resistance measured by four-wire measurement. (b) Box plot of electrode resistance for $R_{electrode\ 1}$. (c) Box plot of electrode resistance for $R_{electrode\ 2}$. (d) Box plot of electrode resistance for $R_{electrode\ 3}$. (e) Schematic of the bonded electrode design with three electrode wires and its equivalent circuit. (f) Box plot of electrode resistance for the bonded sample ($R_{bonded\ electrode}$) measured via four-wire measurement.

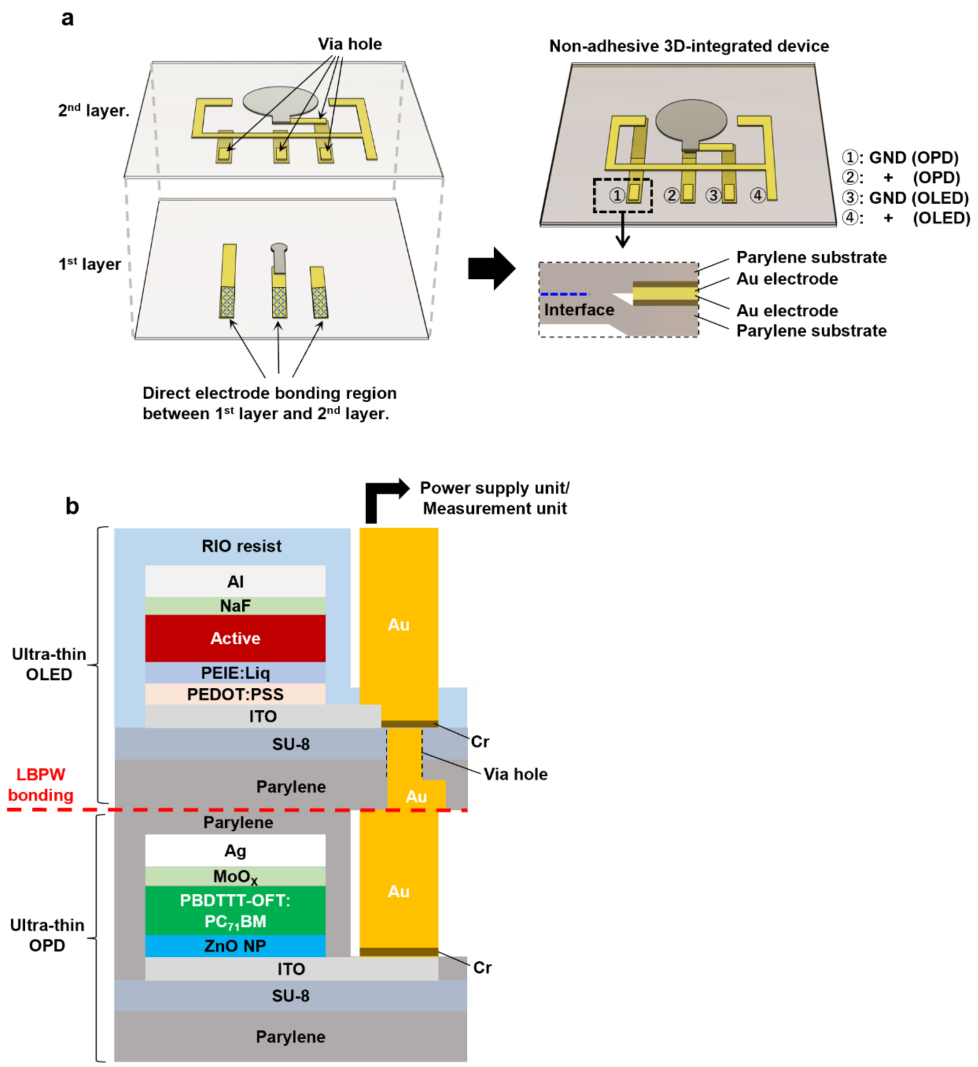


Figure S25. Detailed structure of the 3D-stacked PPG sensor.

(a) Schematic of the ultra-flexible PPG sensor, where the 1st layer is an ultrathin OPD, and the 2nd layer is an ultrathin OLED. The backside electrodes of the 2nd layer and part of the 1st layer are bonded using LBPW to establish conductive connections between layers. LBPW also bonds the parylene substrate layers simultaneously during electrode bonding. (b) Cross-sectional structure of the PPG sensor.

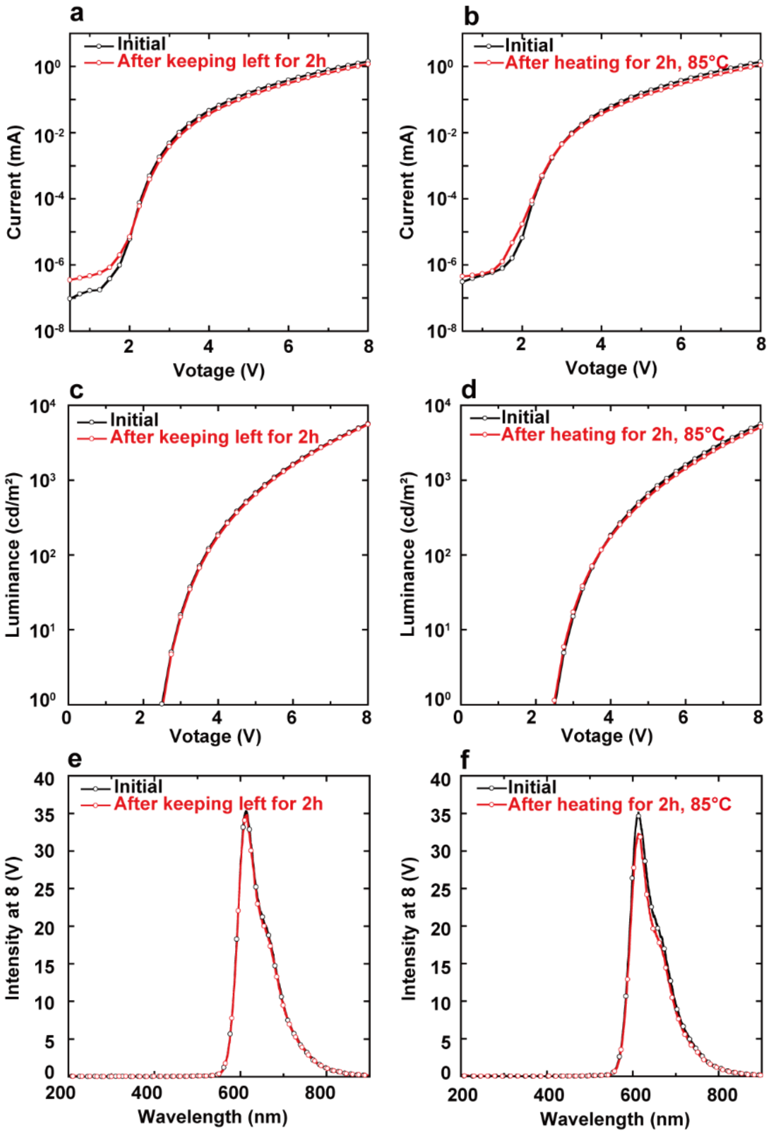


Figure S26. Performance degradation of the OLED in air and after the LBPW process.

(a, b) Changes in current–voltage curves before and after treatment. (c, d) Changes in luminance–voltage curves before and after treatment. (e, f) Changes in intensity–wavelength curves before and after treatment.

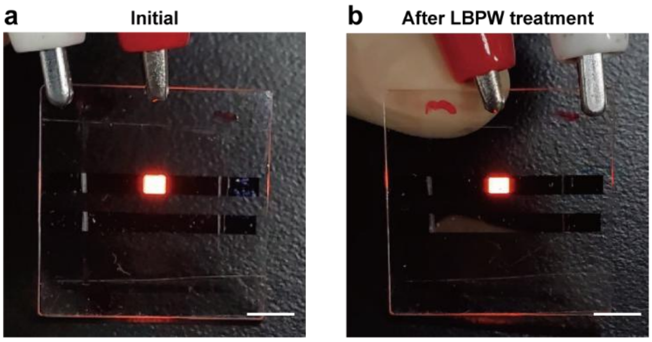


Figure S27. OLED area changes before and after the LBPW process.

(a) Initial state before LBPW treatment. (b) Post-LBPW treatment, following water vapor plasma exposure, water droplet application, and oven heating at 85 °C in an $N_2$ atmosphere for 2 h. All scale bars represent 5 mm.

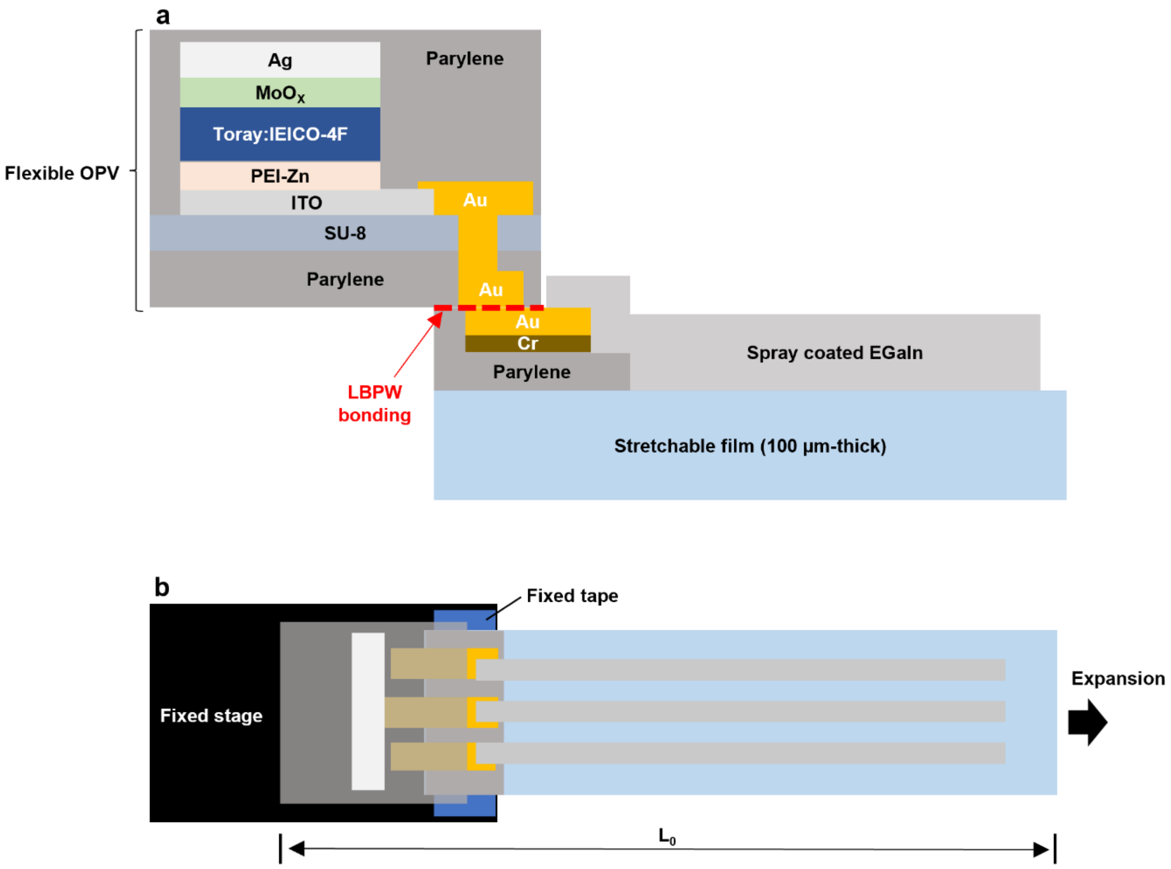


Figure S28. Structure and experimental setup for the stretchable OPV.

(a) Cross-sectional view of the stretchable OPV with LBPW bonding. (b) *J–V* performance measurement of the OPV during expansion.

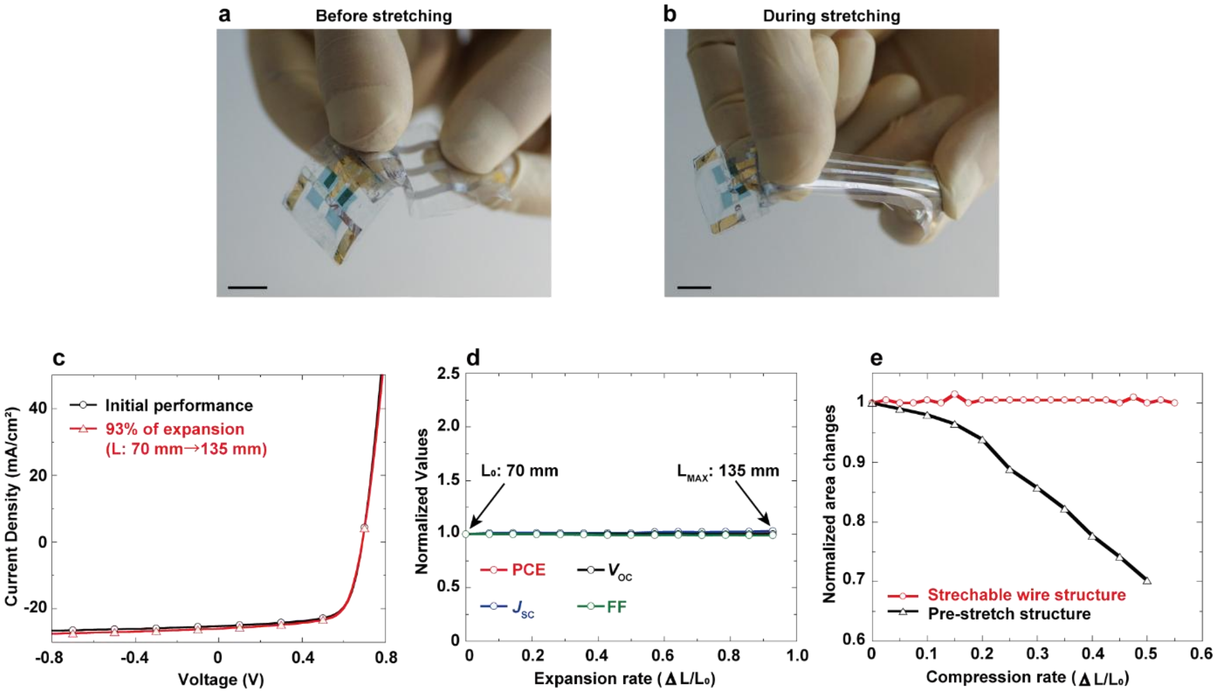


Figure S29. Stretchable OPV fabricated via LBPW bonding.

(a, b) Photographs of the stretchable OPV using LBPW bonding. (a) Initial state. (b) During stretching. (c) *J–V* characteristics comparing the initial state and maximum expansion. (d) Parameter variations of the stretchable OPV under expansion. (e) Comparison of OPV area changes during deformation with pre-stretched OPV structures. All scale bars represent 10 mm.

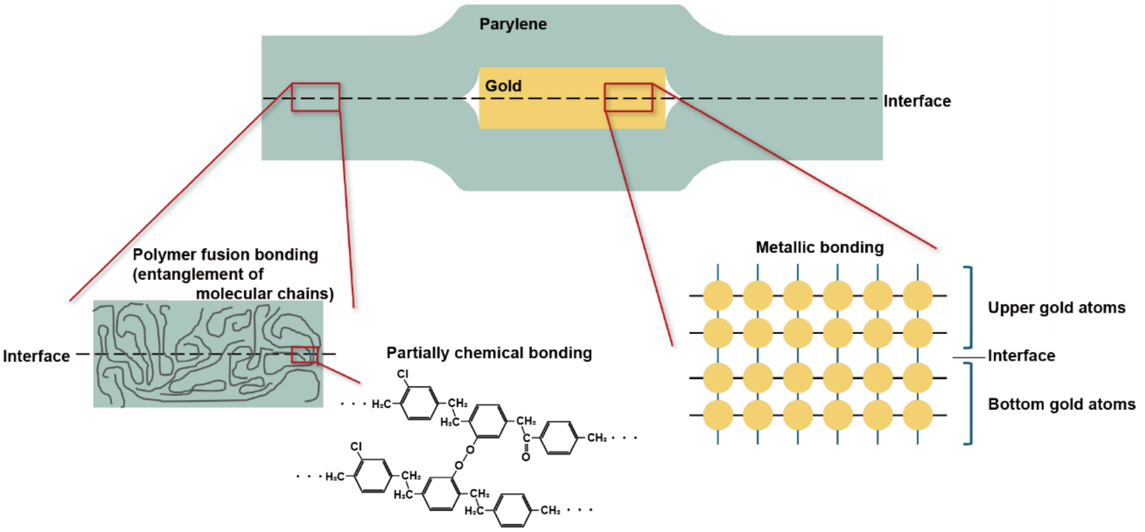


Figure S30. Proposed microscopic bonding mechanism of the LBPW method.
Atomic-level bonding occurs in the Au regions, while molecular chain entanglement and partial chemical bonding are expected in the parylene polymer regions.

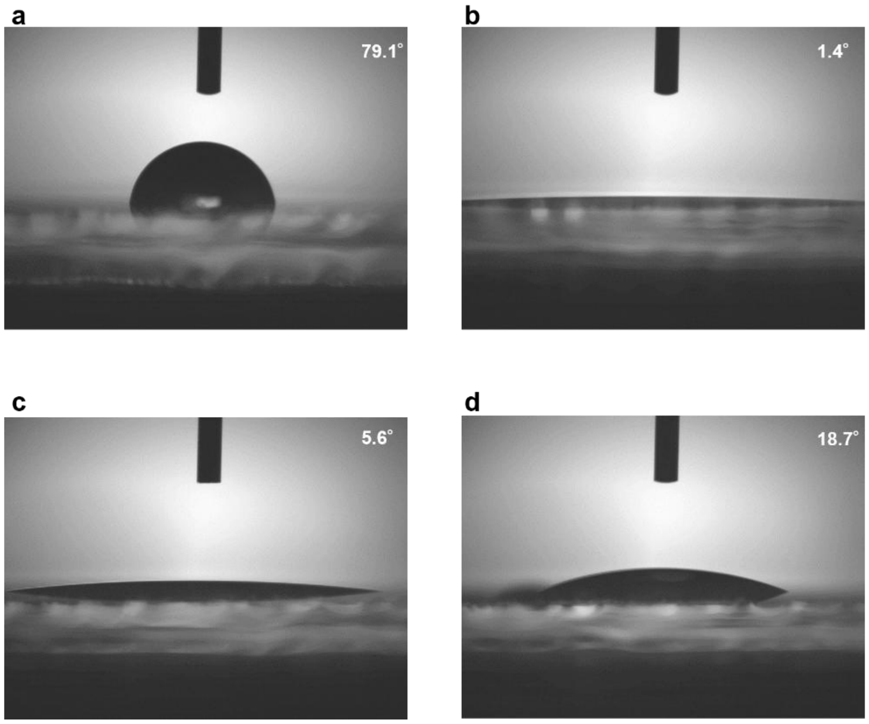


Figure S31. Contact angle measurements of the parylene surface.

(a) Untreated surface. (b) Water-vapor plasma-treated surface. (c) Argon plasma-treated surface. (d) Oxygen plasma-treated surface.

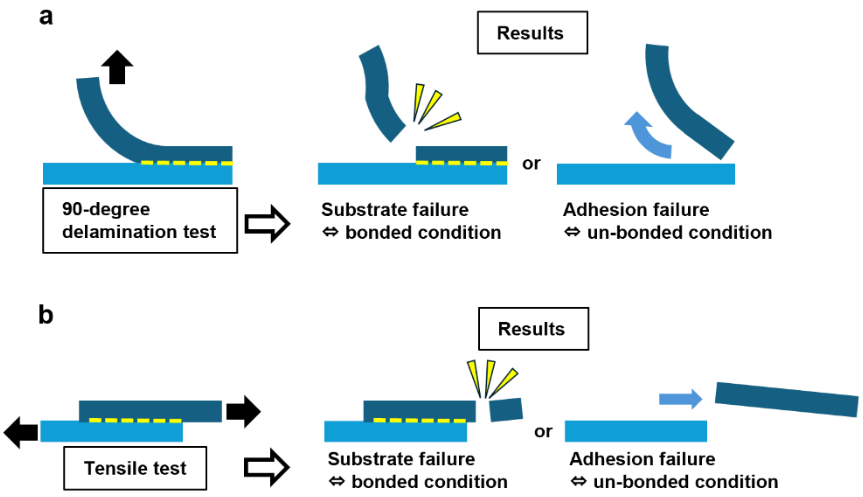


Figure S32. Definitions of bonded/non-bonded states for destructive testing. (a) 90° delamination test. (b) Tensile test.

Table S1. Comparison of LBPW (this work) with previous bonding methods for flexible electronics integration.

| | **LBPW (This work)** | **ACF** [10] | **ACA** [10] | **WVPAB** [18] | **BIND interface** [11] |
|---|---|---|---|---|---|
| **Bonding method of devices** | Direct bonding | Inserting between electronics | Inserting between electronics | Direct electrode bonding | Direct bonding |
| **Conductive method at the interface** | Metallic bond | Contact conductive particle | Contact conductive particle | Metallic bond | Contact metal particle |
| **Process Temperature** | ~85 | RT~200 | RT | RT | RT |
| **Process Pressure** | ~1.5 MPa | 2~5 MPa | 0.1 MPa | ~1.5 MPa | - |
| **Minimum pitch size** | Less than 5 μm | ~ 80 μm | ~ 10 μm | ~10 μm | 100 μm |
| **Conformability (Minimum bending radius)** | ~ 0.5 mm | ~5 mm of bending radius | ~20 mm | ~ 0.5 mm | - |
| **Bondable material** | Parylene polymer, gold metal | Polymer, Metal | Polymer, Metal | Gold only | SEBS |

ACA: Anisotropic Conductive Adhesive
BIND interface: biphasic nano-dispersed interface
RT: Room temperature

Table S2. Bonding performance of parylene coatings with various materials and thicknesses.

All samples maintained their parylene bond, though delamination occurred between the base material and the coated parylene.

| **Substrate A (Thickness)** | **Substrate B (Thickness)** | **The result of the delamination test (Failure mode)** |
|---|---|---|
| PET (1 μm) +Parylene (1.3 μm) | PET (1 μm) +Parylene (1.3 μm) | Delamination from PET/Parylene interface (Substrate failure) |
| Polyimide (5 μm) +Parylene (1.3 μm) | Polyimide (5 μm) +Parylene (1.3 μm) | Delamination from Polyimide/Parylene interface (Substrate failure) |
| PET (1 μm) +Parylene (1.3 μm) | Parylene (2 μm) | Delamination from PET/Parylene interface (Substrate failure) |
| Polyimide (5 μm) +Parylene (1.3 μm) | Parylene (2 μm) | Delamination from Polyimide/Parylene interface (Substrate failure) |

Table S3. IR spectrum peaks detected by FTIR measurement.

| **Absorption peak** [S13] **($cm^{-1}$)** | **Chemical group** |
|---|---|
| 3943 | O–H stretching |
| 3705 | O–H stretching |
| 3468 | O–H stretching |
| 3383 | N–H stretching |

Table S4. Elemental composition of bonded and unbonded surfaces measured by XPS.

Detected peaks for N, Na, Zn, Ca, and Si are likely due to residual matter inside the equipment and are not expected to influence LBPW bonding.

| Signal | BE (eV) | Bonded surface (%) | Unbonded surface (%) |
|---|---|---|---|
| C 1s | 285.0 | 78.7 | 83.9 |
| Cl 2p | 200.7 | 7.8 | 8.3 |
| O 1s | 532.8 | 10.7 | 6.1 |
| N 1s | 400.2 | 0.9 | 0.6 |
| Na 1s | 1072.1 | 0.1 | 0.1 |
| Zn 2p | 1022.4 | 0.3 | 0.2 |
| Ca 2p | 348.1 | 0.2 | 0.2 |
| Si 2p | 102.2 | 1.4 | 0.7 |

Table S5. Curve fitting results for the C 1s XPS spectra on bonded and unbonded surfaces.

| **Name** | **Binding energy (eV)** | **Unbonded region** | **Bonded region** |
|---|---|---|---|
| | | Ratio (%) | Ratio (%) |
| C–H | 285.0 | 73.4 | 69.0 |
| $C_X$–Cl | 286.0 | 13.6 | 19.1 |
| C–O | 286.7 | 6.0 | 3.2 |
| C=O | 287.8 | 1.6 | 2.8 |
| C–O–O | 289.4 | 0.4 | 1.6 |
| Shake-up | 291.5 | 5.0 | 4.2 |

Movie S1. Comparison of bonding stability between hydrogen bonding and LBPW in underwater conditions.

Two types of bonded parylene samples were prepared: one bonded using LBPW and the other hydrogen-bonded by plasma treatment. Both samples were immersed in water. The hydrogen-bonded sample delaminated easily, while the LBPW-bonded sample maintained stable underwater bonding.

Movie S2. Water immersion stability test of LBPW-bonded parylene samples.

Two large parylene films were bonded using LBPW with an air pocket. The bonded samples were immersed in water for eight days. After immersion, the air pocket remained intact, and even after pinching and shaking, no air leakage was observed.

**SI reference**


[S1] D. Ziegler, T. Suzuki, and S. Takeuchi, *J. Microelectromechanical Syst*. **2006**, 15, 1477.

[S2] H. Noh, K. Moon, A. Cannon, P. J. Hesketh, C. P. Wong, *J. Micromech. and Microeng*. **2004**, 14, 625.

[S3] H.-S. Noh, Y. Huang and P. J. Hesketh, *Sens. Actuators B Chem.* **2004**, 102, 78.

[S4] H. Kim, K. Najafi, *J. Microelectromech. Syst.* **2005**, 14, 1347.

[S5] F. Selbmann, M. Baum, C. Meinecke, M. Wiemer, H. Kuhn, Y. Joseph, *J. Solid. State. Chem*. **2021**, 10, 074010.

[S6] V. Maharshi, I. Ahmad, A. Agarwal and B. Mitra, *J. Micromech. Microeng.* **2023**, 33, 014004.

[S7] V. Maharshi, A. S. Khan, A. Agarwal and B. Mitra, *IEEE Trans. Compon. Packag. Manuf. Technol.* **2024**, 14, 194-201

[S8] Q. Shu, X. Huang, Y. Wang, J. Chen, *9th International Conference on Solid-State and Integrated-Circuit Technol.* **2008,** 4735061

[S9] H. kim, K. Najafi, *J. Microelectromech. Syst.* **2007,** 16, 1386

[S10] W. Li, D. C. Rodger, A. Pinto, E. Meng, J. D. Weiland, M. S. Humayun, Y. Tai, *Sensor and Actuators A*, **2011**, 166, 193-200

[S11] Y. Yen, Y. Lee, Y. Chang, W. Fang, *IEEE 25th International Conference on MEMS*, **2012**, 6170213

[S12] H. Noh, P. J. Hesketh, G. C. Frye-Mason, *J. Microelectromechanical Syst.* **2002,** 11, 718.

[S13] Merck, IR spectrum table, https://www.sigma-Aldrich.com/JP/ja/technical-documents/technical-article/analytical-chemistry/photometry-and-reflectometry/ir-spectrum-table? report=reader, accessed July **2023**.
